# Sclerotomal hematopoiesis in vertebrate embryos contributes to robustness of the blood system

**DOI:** 10.1093/nsr/nwag035

**Published:** 2026-01-19

**Authors:** Zheng Jiang, Tianqi Li, Yixiao Song, Luxi Chen, Juhui Qiu, Fan Zhou, Xiaotong Wu, Jianbin Wang, Anming Meng

**Affiliations:** Laboratory of Stem Cell Regulation, Guangzhou National Laboratory, Guangzhou 510320, China; Laboratory of Molecular Developmental Biology, State Key Laboratory of Membrane Biology, Tsinghua University, Beijing 100084, China; Tsinghua-Peking Center for Life Sciences, School of Life Sciences, Tsinghua University, Beijing 100084, China; Tsinghua-Peking Center for Life Sciences, School of Life Sciences, Tsinghua University, Beijing 100084, China; Laboratory of Molecular Developmental Biology, State Key Laboratory of Membrane Biology, Tsinghua University, Beijing 100084, China; Tsinghua-Peking Center for Life Sciences, School of Life Sciences, Tsinghua University, Beijing 100084, China; Laboratory of Molecular Developmental Biology, State Key Laboratory of Membrane Biology, Tsinghua University, Beijing 100084, China; Tsinghua-Peking Center for Life Sciences, School of Life Sciences, Tsinghua University, Beijing 100084, China; Key Laboratory for Biorheological Science and Technology of Ministry of Education, State and Local Joint Engineering Laboratory for Vascular Implants, Bioengineering College of Chongqing University, Chongqing 400030, China; Tsinghua-Peking Center for Life Sciences, School of Life Sciences, Tsinghua University, Beijing 100084, China; Laboratory of Molecular Developmental Biology, State Key Laboratory of Membrane Biology, Tsinghua University, Beijing 100084, China; Tsinghua-Peking Center for Life Sciences, School of Life Sciences, Tsinghua University, Beijing 100084, China; Tsinghua-Peking Center for Life Sciences, School of Life Sciences, Tsinghua University, Beijing 100084, China; State Key Laboratory of Green Biomanufacturing, and Center for Synthetic & Systems Biology, Tsinghua University, Beijing 100084, China; Laboratory of Stem Cell Regulation, Guangzhou National Laboratory, Guangzhou 510320, China; Laboratory of Molecular Developmental Biology, State Key Laboratory of Membrane Biology, Tsinghua University, Beijing 100084, China; Tsinghua-Peking Center for Life Sciences, School of Life Sciences, Tsinghua University, Beijing 100084, China

**Keywords:** hematopoiesis, hematopoietic stem and progenitor cells (HSPCs), sclerotome, somite

## Abstract

During vertebrate embryogenesis, hematopoietic stem cells (HSCs) are believed to almost exclusively arise from hemogenic endothelial cells (ECs) of the dorsal aorta through endothelial-to-hematopoietic transition (EHT). It remains elusive whether HSCs can be generated by other tissues. Sclerotomal cells (SCs) in embryonic somites predominantly form vertebrae and ribs, and some SCs may also insert into aortic endothelia. Here we show that a subset of SCs directly enter the vascular lumen to become hematopoietic stem and progenitor cells (HSPCs) in an EHT-independent way in both zebrafish and mouse embryos. The sclerotome-derived HSPCs (scHSPCs) contribute to various blood cells throughout the lifetime. The proportion of scHSPCs increases dramatically when endothelium hematopoiesis is defective. Thus, the sclerotomal hematopoiesis is evolutionarily conserved and would ensure the robustness of hematopoiesis.

## INTRODUCTION

Embryonic hematopoiesis occurs in two waves: primitive and definitive. Primitive hematopoietic progenitors (PHPs) arise in the mammalian yolk sac (YS) or intermediate cell mass (ICM) and rostral blood island (RBI) in teleost fish, producing short-lived erythrocytes and macrophages [1]. Definitive hematopoietic stem and progenitor cells (HSPCs), being capable of lifelong self-renewal and multilineage differentiation, originate predominantly from endothelial cells (ECs) in the dorsal aorta via endothelial-to-hematopoietic transition (EHT) across vertebrates [2–4].

In 1995, Pardanaud and Dieterlen-Lievre showed that quail somites depleted in aortic ECs might generate some hemogenic cells upon transplantation into chick embryos [5]. In recent years, increasing evidence has proposed that somite stromal cells may generate aortic endothelia [6–8] or hemogenic cells [9–11] through EHT in multiple models. Our previous study in zebrafish also suggests a somitic contribution to HSPCs [12]. However, whether somites can directly contribute to hematopoietic cells without endothelial transition is still a controversy in the stem cell field, due to a lack of direct evidence. It is also unclear which somite compartment is hemogenic. More important questions are how somitic hematopoiesis coordinates with aortic endothelial hematopoiesis and whether it is conserved in mammalian embryos.

In this study, we demonstrate that a subset of cells within the sclerotome of fish and mouse embryos may transdifferentiate into hematopoietic cells mainly independent of EHT and that sclerotomal hematopoiesis could compensate for loss of blood cells due to disruption of endothelial hematopoiesis.

## RESULTS

### Ontogeny of sclerotome-derived hematopoietic cells in zebrafish embryos

**Figure 1.**
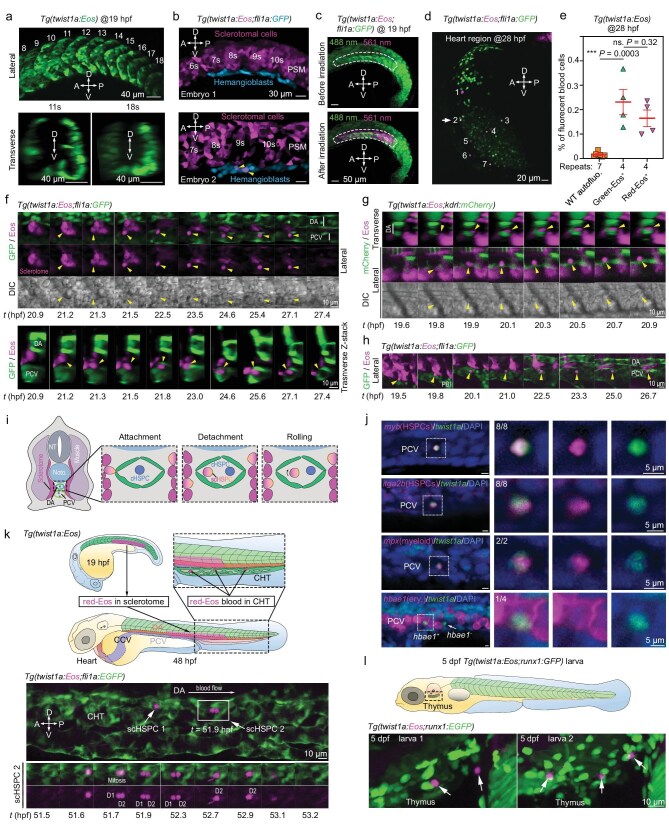
Ontogeny of sclerotome-derived HSPCs in zebrafish embryos. (a) Confocal microscopic images of Eos fluorescence pattern in *Tg(twist1a: Eos)* at 19 hpf. Upper row, lateral view with anterior to the left and somite numbers indicated; lower row, transverse views at the 11th somite and 18th somite levels. The bidirectional arrows indicated the dorsal (D)–ventral (V)–anterior (A)–posterior (P) axis. (b) Two representative embryos showing red-Eos^+^ sclerotomal cells and *fli1a*: GFP^+^ hemangioblasts. The whole trunk of *Tg(twist1a: Eos; fli1a: GFP)* embryos at 12 hpf was UV-irradiated and observed laterally by confocal microscopy at 14 hpf. The somite order is indicated. PSM, presegmental mesoderm. Two double-positive cells in Embryo 2 are indicated by arrows. (c) Images of the trunk region of a representative *Tg(twist1a: Eos; fli1a: GFP)* double-transgenic embryo before and immediately after photoconversion at 19 hpf. (d) Red Eos^+^ blood cells (numbered) in the heart of a 28-hpf *Tg(twist1a: Eos; fli1a: GFP)* embryo, whose somites were UV-irradiated at 20 hpf. Arrow indicates *twist1a*: Eos; *fli1a*: GFP double-positive blood cells. (e) Proportion of fluorescent blood cells at 28 hpf. Green-Eos^+^ or red-Eos^+^ blood cells of untreated or UV-irradiated (at 20 hpf) *Tg(twist1a: Eos)* embryos were analyzed by flow cytometry, respectively. Blood cells of 28-hpf WT embryos were also analyzed for red autofluorescence as control (WT autofluo). Ten to twenty larvae were used for each batch. (f–h) Time-lapse fluorescence imaging of one single sclerotomal cell (indicated by yellow arrowheads) emigrating into the PCV (f), DA (g) or PBI (h). UV-irradiation at 19–20 hpf; Z-stack images; t, timepoints; embryos positioned with the anterior to the left. (i) Schematic diagram of sclerotomal cell emigration (to DA and PCV) process; NT, neural tube. (j) FISH detection of marker expression in single blood cells in PCV at 28 hpf. Right three columns, enlarged images of the insert in the left column; the ratio of double-positive cells to *twst1a*^+^ cells is indicated; ery, erythrocyte. (k) Schematic diagram (top) and live images (bottom) showing observation strategy and division of a red-Eos^+^ scHSPC in CHT, respectively. D1 and D2, daughter cells 1 and 2 of scHSPC 2, respectively. (l) Residence of *twsist1a*: Eos^+^ scHSPCs in thymus region with *runx1*: GFP^+^ blood cells in 5-dpf double transgenic embryos in which 10–11 somites were UV-irradiated at 20 hpf.

Given that sclerotome cells (SCs) adjacent to the aorta are highly migratory [13], they may have a good chance of entering the aortic lumen. To visualize migration of SCs, we used zebrafish *Tg(twist1a: Eos)* transgenic embryos, which express photoconvertible Eos fluorescent protein specifically in the sclerotome during somitogenesis (Fig. 1a), and *Tg(twist1a: Eos; fli1a: GFP)* double transgenic embryos in which lateral plate mesoderm-derived hemangioblasts are labeled by green fluorescent protein (GFP) during early somitogenesis. To confirm *twist1a*: Eos expression specificity, we UV-irradiated all somites to convert green-Eos to red-Eos in *Tg(twist1a: Eos; fli1a: GFP)* double transgenic embryos at 12 h postfertilization (12 hpf) (about 6-somite stage) at which sclerotomal cells have not started migration, and observed/counted fluorescent cells in embryos at 14 hpf (around 10-somite stage) at which some SCs have started to migrate. By confocal microscopy (Fig. 1b), we carefully observed a total of 43 optical sections from 26 embryos, and found only 8 *twist1a*: Eos; *fli1a*: GFP double-positive cells among 6327 *twist1a*: Eos^+^ cells and 1188 *fli1a*: GFP^+^ cells. These eight double-positive cells were most likely derived from *twist1a*: Eos^+^ SCs and were undergoing endothelial transition with activation of *fli1a*: GFP expression. This observation further indicates a sclerotomal specificity of *twist1a*: Eos expression.

Next, we laser-irradiated the ventral part of the middle trunk of *Tg(twist1a: Eos; fli1a: GFP)* embryos at 19–20 hpf (Fig. 1c, Fig. S1a and S1b). Subsequent microscopic observations revealed circulating red-Eos^+^ cells in the heart (Fig. 1d) and the vascular lumen (Fig. S1c). Flow cytometry of blood cells from non-photoconverted or photoconverted *Tg(twist1a: Eos)* embryos at 28 hpf identified 0.23% green-Eos^+^ or 0.17% red-Eos^+^ cells, respectively (Fig. 1e), suggesting a low proportion of sclerotome-derived blood cells at this observation stage. Besides this, some *twist1a*: Eos^+^ cells were found in between the aortic floor and the cardinal vein roof (Fig. S1c) and a few *twist1a*: Eos^+^ cells appeared to be endothelia with co-expression of *flia*1a: GFP (Fig. S1d), being consistent with previous reports that SCs could contribute to perivascular stromal cells and endothelia [7–9]. Interestingly, we noted that, as analyzed by flow cytometry using 28-hpf double-transgenic embryos, none of the *twist1a*: Eos^+^ blood cells was *gata1a*: GFP^+^, but a small portion (<7%) of *twist1a*: Eos^+^ blood cells were also *fli1a*: GFP^+^ or *kdrl*: mCherry^+^ (Fig. S1e), and that *twist1a*: Eos; *fli1a*: GFP double-positive blood cells were occasionally observed by confocal microscopy (Fig. 1d, indicated by arrow). These observations imply that most of the *twist1a*: Eos^+^ blood cells should be generated from SCs through direct sclerotome-to-hematopoietic transition and a few *twist1a*: Eos^+^ blood cells may have undergone EHT.

We then carefully observed the sclerotome-to-hematopoietic transition process of SCs in living *Tg(twist1a: Eos; fli1a: GFP)* or *Tg(twist1a: Eos; fli1a: GFP)* embryos that were UV-irradiated in ventral somites at 19–20 hpf. We found that a single cell within a red-Eos^+^ SC cluster could migrate medially to attach to and penetrate the inter-endothelial junction of the posterior cardinal vein (PCV) (Fig. 1f and Video S1) or dorsal aorta (DA) (Fig. 1g), or to cram into the posterior blood island (PBI) (Fig. 1h), and then started to roll in the circulation, which generally involved three steps (Fig. 1i). Based on live image tracking of 30 sclerotome-derived blood cells, the ontogeny of such a cell took 2–10 h (Fig. S1f), which was comparable to that for the emergence of endothelium-derived HSPCs (eHSPCs) in the zebrafish [2,14]. We were unable to capture any SCs that first transformed into endothelia and then differentiated into blood cells, likely because this event is infrequent. As analyzed below, SC-derived circulating blood cells have characteristics of HSPCs and are thus referred to as scHSPCs.

Fluorescent *in situ* hybridization (FISH) in 28–36 hpf embryos detected co-expression of *twist1a* with the hematopoietic stem cell markers *cmyb* [14] or *itga2b*/*CD41* [15] in individual scHSPCs (Fig. 1j), supporting their sclerotomal origin. Some scHSPCs in the circulation co-expressed *twist1a* with the neutrophil progenitor marker *mpx* or erythrocyte gene *hbae1* (Fig. 1j), suggesting their differentiation. Tracking of red-Eos^+^ scHSPCs revealed that they resided in the vein plexus of the caudal hematopoietic tissue (CHT) for 53.18 min on average (Fig. S1g), during which they might undergo cell division (Fig. 1k, Video S2). Some scHSPCs at 5 days postfertilization (5 dpf) could be found in the thymus (Fig. 1l), a later hematopoietic niche. These observations indicate that scHSPCs, after entering the circulation, may colonize the hematopoietic tissues in a way similar to eHSPCs [16].

### Distinct transcriptional characteristics of zebrafish scHSPCs

To better understand hematopoietic cell types during development, we performed 10× Genomics single-cell RNA-seq (scRNA-seq) of whole blood at 28 hpf, 56 hpf and 7 dpf, peripheral blood (PB) without red blood cells (RBCs) at 1 month postfertilization (1 mpf) and 2.5 mpf, as well as whole kidney marrow (WKM) at 2.5 mpf. After standard quality filtering and removal of infiltrated RBCs with high-level expression of hemoglobin genes such as *hemgn*, as well as non-hematopoietic epithelium and neuron cells, a total of 6167 blood cells were selected for clustering analysis. This analysis identified four clusters, including HSPCs, lymphoid, myeloid and macrophage cells (Fig. S2a). HSPCs could be further clustered into two subpopulations, the scHSPC subpopulation with preferred expression of many hematopoietic genes (e.g. *meis1b* [17,18], *cebpd* [19], *tcf12* [20] and *id1* [21,22]), as well as somite/skeletal/muscle genes, and the classical HSPCs (cHSPCs) with hematopoietic markers but without somite/skeletal/muscle markers (Fig. S2b–g). Hematopoietic gene expression in scHSPCs was confirmed by FISH (Fig. S2h).

Next, we sorted *twistl1a*: Eos^+^ cells (scHSPCs) from blood at 28, 36, 46, 52, 72 and 96 hpf. For tracing the lineage commitment of scHSPCs, several types of cells from different transgenic embryos were also used: *twist1a*: Eos^+^ SCs at 19 hpf; *tal1*: GFP^+^ primitive hematopoietic precursors (PHPs) at 19 hpf [23]; *kdrl*: mCherry^+^; *runx1*: GFP^+^ eHSPCs at 28, 36, 52 and 72 hpf [24]; *coro1a*: GFP^+^ macrophages at 28 hpf [25]; and *mpx*: GFP^+^ neutrophils at 28 hpf [26]. Because of limited cell numbers, we performed single-cell RNA-seq using Smart-seq2 (Table S1), which was demonstrated to have higher sensitivity and precision [27].

**Figure 2.**
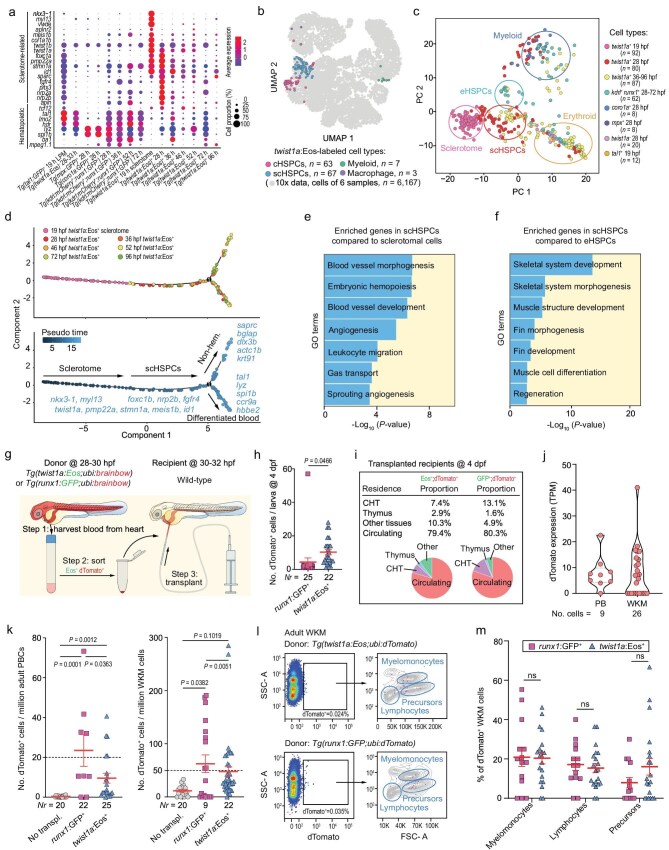
Molecular characterization of zebrafish sclerotome-derived HSPCs. (a) Expression of hematopoietic and sclerotome-related gene sets in Smart-seq2-analyzed blood cells from different transgenic lines. (b) Projection of *twist1a*: Eos^+^ cells to the integrated UMAP of WT blood cells (see Fig. S2b). (c) PCA of blood cells from different transgenic lines, identifying four major populations. (d) Pseudo-time trajectory analysis of transgenic *twist1a*: Eos^+^ cells (top) with indicated markers (bottom). (e and f) GO term analysis of upregulated genes in scHSPCs. (g) Transplantation strategy and procedure. (h) Number of viable transplanted dTomato^+^ cells per recipient at 4 dpf as counted by microscopy. *Nr*, number of observed recipients. (i) Estimated blood contribution of donor cells in recipients by microscopic counting at 4 dpf. (j) dTomato expression levels of dTomato^+^ cells sorting from PB or from WKM of adult recipients, analyzed by Smart-Seq2. (k) Estimated number of donor-derived blood cells in adult recipient PBCs (left) or WKM (right) by fluorescence-activated cell sorting (FACS); *Nr*, number of analyzed recipients; two-tailed *t*-test was used with *P* values indicated. (l and m) Representative flow cytometry results of dTomato^+^ blood cells in WKM of adult recipients (l) and proportions of different cell types among dTomato^+^ WKM cells (m) in different individuals. Fourteen recipients transplanted with *runx1*: GFP^+^ donor cells and 20 recipients transplanted with *twist1a*: Eos^+^ donor cells were analyzed. Two-tailed *t*-test was performed. ns, non-significant.

Smart-seq2 profiling revealed distinct expression patterns in respect to hematopoietic and sclerotomal genes in different sources of cells (Fig. 2a). When projected to the integrated UMAP based on 10× Genomics blood cell scRNA-seq data (Fig. S2a), 47.9% (67/140) and 45.0% (63/140) of *twist1a*: Eos^+^ blood cells from 28 to 96 hpf fell into the scHSPC cluster and the cHSPC cluster (Fig. 2b), respectively. Those scHSPCs falling into the cHSPC cluster might result from their differentiation with loss of somite/skeletal/muscle markers at some stages. Principal component analysis (PCA) of all cells analyzed by Smart-seq2 could identify five major cell clusters: SCs, scHSPCs, eHSPCs, myeloid cells and erythroid cells (Fig. 2c), which well reflected their sources. Notably, *twist1a*: Eos*^+^* SCs at 19 hpf were close to but separated from *twist1a*: Eos*^+^* blood cells at 28 hpf along PC1, suggesting differentiation tendency from SCs to blood cells. The majority of sclerotome-derived *twist1a*: *Eos^+^* blood cells neither express the primitive hematopoietic marker genes *tal1(scl), lmo2* and *gata1* nor express the endothelial genes *fli1a* and *kdrl* (Fig. S2i), suggesting their distinct origination from classical hematopoietic cells. The *twist1a*: Eos^+^ blood cells from 36 hpf onwards and *kdrl*: mCherry^+^; *runx1*: GFP^+^ eHSPCs were apparently separated along two directions: one towards the cluster containing *coro1a*: GFP^+^ and *mpx*: GFP^+^ myeloid cells, and the other towards the population with erythrocyte gene expression. Thus, both scHSPCs and eHSPCs may gain differentiation capacity either to erythroid fate or myeloid fate. Pseudo-time ordering by trajectory analysis of *twist1a*: Eos^+^ cells from 19 to 96 hpf indicated that SCs initially differentiated into scHSPCs, and then branched along two major trajectories: hematopoietic fate or nonhematopoietic fate (Fig. 2d). Non-hematopoietic differentiation potential of scHSPCs was supported by a transplantation experiment (see below). Compared to undifferentiated SCs, scHSPCs showed enrichment for blood vessel development and embryonic hematopoiesis (Fig. 2e). Compared to eHSPCs, however, scHSPCs had enriched transcripts for skeletal and muscle development (Fig. 2f). Thus, scHSPCs are distinct from SCs and eHSPCs at the transcriptome level.

### Transplanted zebrafish scHSPCs have self-renewal and multiple differentiation abilities

To check long-term functions by transplantation, we sorted out Eos^+^; dTomato^+^ scHSPCs or GFP^+^; dTomato^+^ eHSPCs from *Tg(twist1a: Eos; ubi: brainbow)* or *Tg(runx1: GFP; ubi: brainbow)* double-transgenic embryos, respectively, in which dTomato can express for the lifetime to allow long-term tracking. Approximately 10–100 sorted cells were transplanted into the common cardinal vein (CCV) of each wildtype (WT) embryo (Fig. 2g). As counted by fluorescent microscopy at 2 days post-transplantation (about 4 dpf), most recipients each had fewer than 20 viable transplanted cells (Fig. 2h), implying that many transplanted cells died or were damaged during transplantation. At this stage, donor scHSPCs or eHSPCs could be observed mostly in the circulation and also in the hematopoietic niche CHT and thymus of recipients (Fig. 2i), suggesting that scHSPCs and eHSPCs have similar abilities to colonize hematopoietic tissues.

When recipients grew up to the adult stage (3–6 months), single-cell sequencing of sorted dTomato^+^ blood cells from PB and WKM confirmed *dTomato* gene expression (Fig. 2j), indicating long-term survival of donor cells. We estimated the number of donor scHSPCs or donor eHSPCs in PB and WKM of each adult recipient by flow cytometry (Fig. 2k). Assuming that an adult fish has about 10 million PB cells (PBCs) and 5 million WKM cells, the estimated total number of donor scHSPC-derived or donor eHSPC-derived blood cells per recipient adult, including those in PB and WKM, should be 333.7 or 546.55, respectively, which meant about a 32-fold or 125-fold increase compared to donor cell number at 4 dpf. Apparently, transplanted scHSPCs and eHSPCs both possess self-renewal capacity.

Flow cytometry analysis of WKM cells of adult recipients indicated that transplanted scHSPCs and eHSPCs contributed to various types of blood cells, including hematopoietic precursors, as well as myeloid and lymphoid cells, with comparable percentages (Fig. 2l and m). Smart-seq2 scRNA data of dTomato-expressing WKM cells from recipient fish and GFP^+^ WKM cells from *Tg(itga2b: GFP)* transgenic adult fish [28] also identified similar blood cell types with distinct marker sets (Fig. S3a and b, Table S2). Besides this, we found that 7 or 11 out of 153 scHSPC recipients from 20 dpf to 19 mpf had dTomato^+^ fin or muscle, respectively, some of which were confirmed by Smart-seq2 scRNA data or PCR (Fig. S3c–l, Table S3). In contrast, we found no dTomato^+^ fin in 104 observed eHSPC recipients and saw one muscle-like cell in those recipients, the latter of which might have been derived from contaminated muscle stem cells in donors. These data together suggest that scHSPCs generally have the potential to differentiate into multiple hematopoietic lineages and at least some scHSPCs may also have non-hematopoietic differentiation potential.

### Zebrafish sclerotomal and endothelial hematopoiesis back each other up

**Figure 3.**
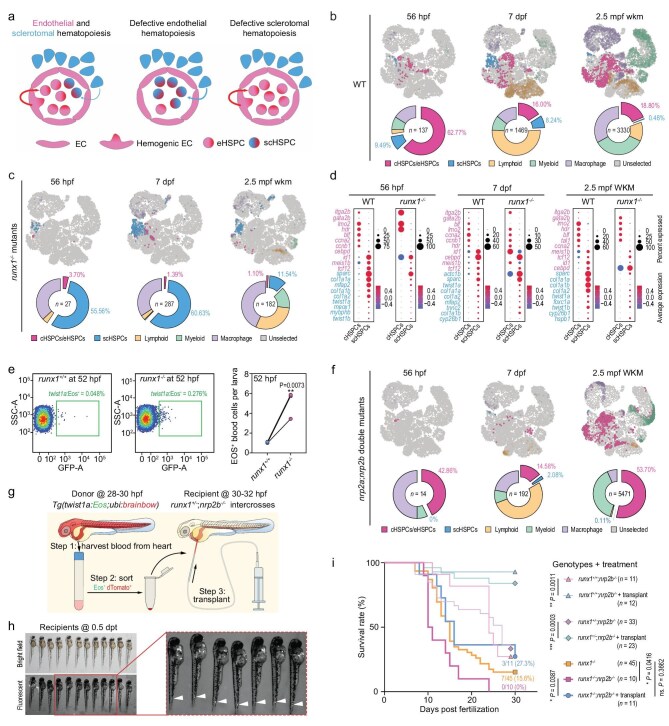
Compensatory effect of sclerotomal and endothelial hematopoiesis in zebrafish. (a) Hypothesis about compensatory effect of two hematopoietic systems. (b and c) Comparison of blood composition between WT and *runx1* mutants; the whole blood taken from WT and *runx1* mutants were subjected to 10× Genomics scRNA-seq, and the remaining cells after filtering RBCs and potential neuronal and epithelial cells were projected to WT 10× Genomics scRNA-seq data (Fig. 2b); UMAP clustering of cells is shown in the top and proportions of blood types are shown in the bottom. (d) Bubble plot of hematopoietic genes (red) and somite-related genes (blue) expression in cHSPCs and scHSPCs of WT organisms and *runx1* mutants. (e) Representative flow cytometry results of Eos^+^ blood cells in *Tg(twist1a: Eos); runx1* mutants (middle panel) or siblings (left panel) at 52 hpf and estimation of *twist1a*: Eos^+^ scHSPCs number in *runx1* mutants and in siblings by flow cytometry (right panel). Three batches of embryos were genotyped at 24 hpf, and blood of 10–20 52-hpf larvae with identical genotype per batch were used for flow cytometry. Unpaired *t*-test was performed. (f) Composition of blood cells of *nrp2a^−^^/^^−^; nrp2b^−^^/−^* double mutants; UMAP clustering of cells is shown in the top and proportions of blood types are shown in the bottom; the projection scores were higher than 0.8; *n*, number of cells with different colors for different types; note that blood cell number in the double mutants at 56 hpf was extremely low. (g) Illustration of scHSPC transplantation. Recipient embryos from intercrosses of *runx1^+/^^−^; nrp2b^−^^/^^−^* fish included three genotypes: *runx1^+/+^; nrp2b^−^^/^^−^, runx1^+/^^−^; nrp2b^−^^/^^−^* and *runx1^−^^/^^−^; nrp2b^−^^/^^−^*. (h) Representative bright and fluorescent images of recipients at 0.5 days post transplantation (dpt). White arrowheads in the right panel indicated accumulated dTomato^+^ blood cells in CHT. (i) Estimation of rescue effect of transplanted scHSPCs on survival rates of larvae with different genotypes. Healthy recipients were picked up about 1 week post-transplantation and observed for survival daily until 30 dpf. Any individuals that died during the observation period and survivors at the end of observation period were genotyped. The significance between lines was statistically tested using the log-rank (Mantel–Cox) test. The *runx1^−^^/^^−^* mutants were obtained from crosses between *runx1* heterozygous fish.

We hypothesize that sclerotomal and classical/endothelial hematopoietic systems may compensate for loss of either one (Fig. 3a). To test this hypothesis, we first analyzed blood cell composition (exclusive of RBCs) in *runx1* mutants at 56 hpf, 7 dpf and 2.5 mpf, the majority (about 80%–85%) of which died at larval stages due to deficiency of eHSPCs, with the remaining surviving to adulthood [29,30], by 10× Genomics scRNA sequencing. Integrated clustering showed that, compared to WT (Fig. 3b), the proportion of scHSPCs increased significantly in *runx1* mutants at all examined stages, which was accompanied by remarkable decrease of the cHSPC/eHSPC proportion (Fig. 3c). Interestingly, the proportion of macrophages in *runx1* mutants also expanded. In addition, the expression of hematopoietic genes and somite-related genes in cHSPCs or scHSPCs of WT and *runx1* mutants generally remained similar (Fig. 3d). Furthermore, flow cytometry analysis also revealed an increase in the number of Eos^+^ blood cells in *Tg(twist1a: Eos); runx1^−^^/^^−^* larva at 52 hpf compared to that in *Tg(twist1a: Eos); runx1^+/+^* siblings (Fig. 3e). These observations together support the idea that loss of eHSPCs, even though not completely, may evoke an expansion of sHSPCs to meet the blood requirement of life.

To specifically remove scHSPCs, we tried to look for potential markers/regulators of scHSPCs. We found that the neuropilin gene *nrp2b* was highly expressed in emerging scHSPCs during sclerotome-to-hematopoietic transition (Fig. 2d). Previous studies revealed expression of *nrp2a* and *nrp2b* in sclerotome/somite and other tissues [31]. Our scRNA-seq data and FISH also detected their expression in the sclerotome and some blood cells (Fig. S4). Since both *nrp2a* and *nrp2b* expression levels were much higher in 28-hpf *twist1a*: Eos^+^ blood cells than in *runx1*: GFP^+^ or *kdrl*: mCherry^+^ blood cells (Fig. S4a and S4b), *nrp2a* and *nrp2b* may be implicated in sclerotomal hematopoiesis specifically. We then created *nrp2a* and *nrp2b* mutant lines in *Tg(twist1a: Eos)* transgenic background using CRISPR/Cas9 technology (Fig. S5a). Flow cytometry showed that *twist1a*: Eos^+^ blood cells at 56 hpf decreased by 53.7% in *nrp2a* mutants, by 50% in *nrp2b* mutants, and by 81.3% in *nrp2a; nrp2b* double mutants (Fig. S5b), which substantiated their roles in sclerotomal hematopoiesis. We did not see obvious morphological defects in somites and fins of *nrp2a*/*nrp2b* single or double mutants (Fig. S5c and S5d). 10× Genomics scRNA-seq analysis indicated that, compared to the ratio of scHSPCs in WT larva/fish (Fig. 3b), this ratio in the *nrp2a; nrp2b* double mutants decreased drastically (Fig. 3f). Unlike in WKM of *runx1* mutants with expanded immune cell populations and a decreased myeloid population (Fig. 3c), interestingly, WKM of *nrp2a; nrp2b* double mutants had an increased myeloid population and a decreased lymphoid cell population (Fig. 3f). This difference may reflect potential distinct and biased differentiation preferences of cHSPCs and scHSPCs. On the other hand, the ratio of cHSPCs in *nrp2a; nrp2b* double mutants is generally comparable to that in WT at 56 hpf and 7 dpf, and increased by nearly 3-fold in WKM at 2.5 mpf compared to WT (Fig. 3b and f). These observations suggest that deficiency of scHSPCs in adults causes a compensatory expansion and biased differentiation of cHSPCs.

To further investigate the biological significance of sclerotomal hematopoiesis, we generated *runx1; nrp2b* double mutants and designed rescue transplantation (Fig. 3g and h). Surprisingly, none of *runx1; nrp2b* double mutants survived beyond 24 dpf, contrasting with about 15% survival rate of *runx1* mutants at 30 dpf (Fig. 3i), which supports the idea that scHSPCs are necessary reinforcements of cHSPCs. We then transplanted Eos^+^; dTomato^+^ scHSPCs into half of the 31-hpf embryos derived from intercrosses between *runx1^+/^^−^; nrp2b^−^^/^^−^* fish (Fig. 3g and h) and used the remaining half as the non-transplanted control group. Results indicated that the 30-dpf survival rates of *runx1^−^^/^^−^; nrp2b^−^^/^^−^, runx1^+/^^−^; nrp2b^−^^/^^−^* and *runx1^+/+^; nrp2b^−^^/^^−^* larvae with transplanted scHSPCs were all significantly increased (Fig. 3i). For instance, 27.3% (3/11) of *runx1^−^^/^^−^; nrp2b^−^^/^^−^* larvae with transplanted scHSPCs still survived at 30 dpf, which was even slightly higher than the survival rate of *runx1^−^^/^^−^* single mutants. This observation supports the idea that scHSPCs are functional and the increased number of scHPCS suggests viability of some *runx1* mutants.

### Hematopoietic transformation of mouse Pax1^+^ SCs

**Figure 4.**
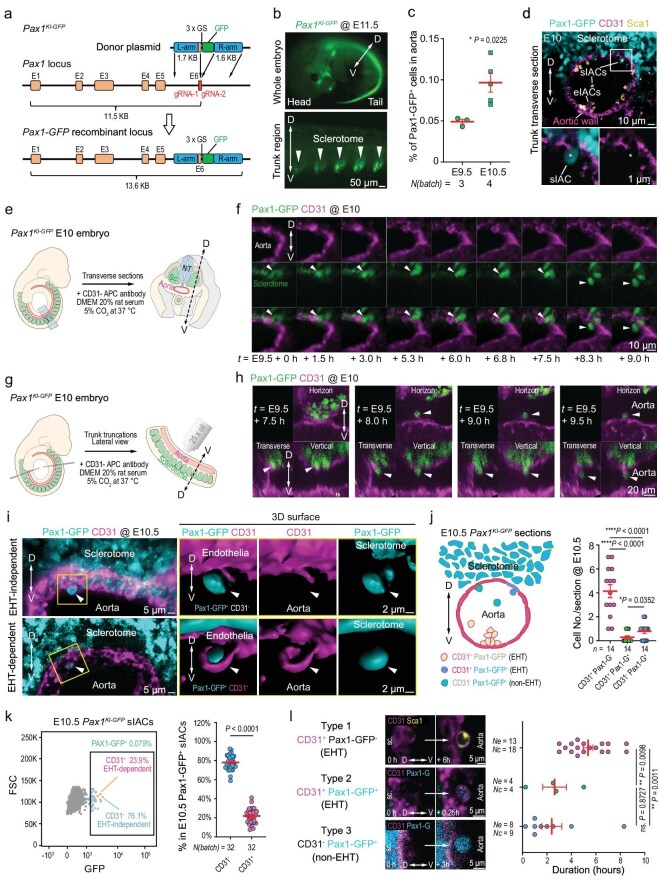
Observation of Pax1-GFP^+^ sclerotomal cell migration towards the aortic lumen in mouse embryos. (a) Illustration of *Pax1-GFP* knock-in strategy. (b) Representative confocal images showing GFP expression in the sclerotome of a heterozygous *Pax1^KI-GFP^* embryo at E11.5. The bidirectional arrow indicates the dorsal (D)–ventral (V) axis (also in the other panels). (c) Percentage of Pax1-GFP^+^ sIACs of E9.5 and E10.5 embryos; IACs were isolated from 3–5 embryos for flow cytometry. Several batches [N(batch)] were done and a two-tailed *t*-test was performed. (d) Cross-section showing Pax1-GFP^+^ cells in an E10 *Pax1^KI-GFP^* embryo stained with CD31-APC antibody (purple) and Sca1-PE (yellow). The boxed area is enlarged below. sIACs and eIACs, putative SC- and endothelial-derived IACs, respectively. The star-indicated cell is a sclerotomal cell just entering the aortic lumen. (e–h) Sectioning and observation strategies (e and g) and time-lapse images (f and h) of Pax1-GFP^+^ SCs migration into the aortic lumen. NT, neural tube; scle, sclerotome. E10 embryos were sectioned and cultured *in vitro*. Observation starting time (*t*) was indicated. A single SC migrating into the aorta lumen was indicated by an arrowhead. (i) Representative live images showing the entry of SCs into the aortic lumen in an EHT-independent (upper panel, CD31^−^) or an EHT-dependent (lower panel, CD31^+^) way in *Pax1^KI-GFP^* embryo sections co-stained with GFP and CD31 antibodies. (j) Ratios of different IACs types; cell numbers were counted using sections from E10–E11 embryos during *ex utero* live imaging; *n*, number of observed sections; two-tailed *t*-test was performed. (k) Representative result of flow cytometry of IACs isolated from E10.5 *Pax1^KI-GFP^* embryos after CD31-APC immunostaining (left) and the average percentage of CD31^−^ Pax1-GFP^+^ or CD31^+^ Pax1-GFP^+^ populations from 32 batches of flow cytometry analyses (right). For each analysis, 3–6 embryos were used. (l) Running time for single SCs or ECs to enter the aortic lumen; left panel shows representative start timepoint and finish timepoint; right panel, statistical results. Type 2 representative image at 0.25 h is the same image in the bottom panel in (i). *Ne*, number of observed embryos (at E10–E10.5); *Nc*, number of observed cells; two-tailed *t*-test was performed.

We wondered whether sclerotomal hematopoiesis is conserved in mammals. To address this issue, we generated a mouse *Pax1^KI-GFP^* line by inserting the GFP coding sequence in frame into the sixth exon of the *Pax1* locus (Fig. 4a), which expressed GFP specifically in the sclerotome in embryos during somitogenesis (Fig. 4b) [32]. Flow cytometry of isolated intra-aortic cells (IACs) indicated that Pax1-GFP^+^ IACs, hereafter referred to as sclerotome-derived IACs (sIACs), accounted for 0.05% and 0.1% of IACs at E9.5 and E10.5, respectively (Fig. 4c). Immunofluorescence on sections of the *Pax1^KI-GFP^* somite region at E10 clearly detected sIACs and presumably endothelial-derived IACs (eIACs) (Fig. 4d). Unlike eIACs often present in the cluster, sIACs looked well separated. We did not detect GFP expression from E8.5 to E10.5 in the YS, an extra-embryonic hematopoietic tissue, by confocal microscopy and RNA sequencing (Fig. S6a and S6b). These observations suggest that Pax1-GFP^+^ SCs might have migrated into the aortic lumen and committed to the hematopoietic fate.

We performed live imaging of *ex utero* sections of *Pax1-GFP* transgenic embryos with CD31-labeled vasculature around E10 and observed migration of some SCs directly into the aortic lumen without endothelial transition (Fig. 4e–h), which is similar to hematopoietic transformation of zebrafish SCs. Interestingly, our time-lapse observations indicated that SCs usually passed through the dorsal and dorsolateral wall of the aorta. Careful microscopic examination identified three types of intra-aortic blood cells: CD31^−^ Pax1-GFP^+^ sIACs, which likely arose from SCs without EHT; CD31^+^ Pax1-GFP^+^ sIACs, which may also arise from SCs but via EHT; and CD31^+^ Pax1-GFP^−^ eIACs derived from aortic hematogenic ECs via EHT (Fig. 4i and j). Combined with flow cytometry results, the proportion of each type was ordered as CD31^+^ Pax1-GFP^−^ > CD31^−^ Pax1-GFP^+^ > CD31^+^ Pax1-GFP^+^ (Fig. 4j and k). Thus, hematogenic SCs may transdifferentiate into blood cells mainly via the EHT-independent process. Time-lapse recording disclosed that the process of the sclerotomal-to-sIAC event was faster (2–3 h) than that (>5 h) of the other two events (Fig. 4l).

### Preferential erythroid and macrophage differentiation of Pax1^+^ hematopoietic cells in mouse embryos

**Figure 5.**
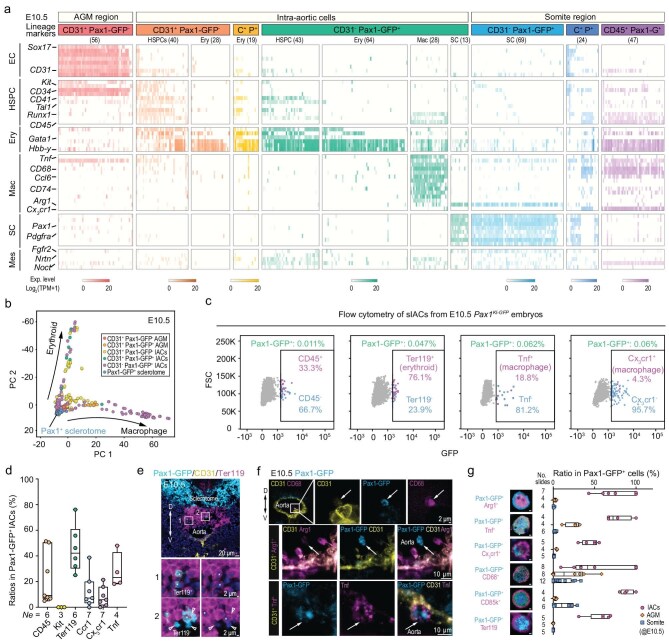
Molecular characterization of mouse embryonic sclerotome-derived hematopoietic cells. (a) Heatmap showing gene expression levels in individual cells of different origins from E10.5 *Pax1^KI-GFP^* embryos based on scRNA-seq data; C^+^ P^+^, CD31^+^ Pax1-GFP^+^; Ery, erythrocyte; Mac, macrophage; Mes, mesenchymal cell; the number of analyzed cells is indicated in parenthesis. (b) PCA of cells with different markers from different locations of E10.5 embryos. (c) Representative flow cytometry results of E10.5 *Pax1^KI-GFP^* sIACs immunostained with CD45-APC, TER119-APC, TNF-APC or CX3CR1-AF647 antibody; the proportion of marker-positive IACs in Pax1-GFP^+^ population is shown. (d) Flow cytometry results of Pax1-GFP^+^ sIACs from E10.5 embryos immunostained with various markers; *Ne*, number of embryos used for assay. (e and f) Representative confocal microscopic images showing signals of indicated markers in Pax1-GFP^+^ sIACs of E10.5 *Pax1^KI-GFP^* embryos on sections. The arrowheads and asterisks in (e) denote Ter119^−^ Pax1^+^ and Ter119^+^ Pax1^+^ cells, respectively; the arrows in (f) indicate Pax1^+^ cells with different macrophage markers. The bidirectional arrow indicates the dorsoventral axis. (g) Ratio of cells with an indicated lineage marker in Pax-GFP^+^ cells; single cell smears of two batches of multiple embryos were immunostained and positive cells were counted on slides.

We sorted CD31^−^ Pax1-GFP^+^ sIACs, along with CD31^−^ Pax1-GFP^+^ SCs, CD31^+^ Pax1-GFP^−^ aorta–gonad–mesonephros (AGM) cells and eIACs, CD31^+^ Pax1-GFP^+^ sIACs, and somite cells from E10.5 *Pax1^KI-GFP^* embryos for Smart-seq2 scRNA sequencing and detected a distinct gene expression pattern for each identified group (Fig. 5a, Table S4). Specifically, CD31^−^ Pax1-GFP^+^ sIACs (148 cells) could fall into four groups: HSPCs (29.1%), erythrocytes (43.2%), macrophages (18.9%) and SCs (8.8%). Those SCs present in sIACs might have been accidentally collected in the IAC samples during tissue dissection. The CD31^−^ Pax1-GFP^+^ HSPCs differed from AGM-derived CD31^+^ Pax1-GFP^−^ HSPCs mainly in two aspects: rare expression of endothelial markers such as *Cd31* and *Sox17*, and lower levels of the HSPC markers *Kit, Cd34* and *Cd45*. Besides this, the majority of CD31^−^ Pax1-GFP^+^ HSPCs expressed erythrocyte markers such as *Gata1* and *Hbb-y*, together with many hematopoietic markers, implying that they are undergoing differentiation. PCA analysis revealed a continuous differentiation trajectory from the sclerotome to either erythrocyte or macrophage lineages (Fig. 5b). Unlike Pax1-GFP^−^ HSPCs or erythrocytes, Pax1-GFP^+^ erythrocytes showed significantly higher levels of both embryonic and definitive hemoglobin genes (Fig. S6c and S6d) [33], suggesting a primitive erythrocyte identity. Pax1-GFP^+^ macrophages exhibited relatively high expression of *Tnf* and *Ccr1*, which were distinguished from somite-resident Cx_3_cr1^+^ macrophages generated from other hematogenic tissues [34]. These data indicate that sclerotome-derived HSPCs may possess erythrocyte/macrophage differentiation bias during embryonic hematopoiesis.

Flow cytometry of E10.5 Pax1-GFP^+^ sIACs using lineage-specific surface makers showed that CD45^+^ Pax1-GFP^+^ cells accounted for only one-third of total Pax1-GFP^+^ cells (Fig. 5c). Those CD45^−^ Pax1-GFP^+^ sIACs were most likely erythrocytes since differentiated erythrocytes do not express CD45 [35], which was supported by the observation that more than half of Pax1-GFP^+^ sIACs had the Ter119 marker (Fig. 5c and d), a marker for differentiated erythrocytes [35]. Approximately one-third of Pax1-GFP^+^ cells were Tnf^+^, but few Pax1-GFP^+^ Cx_3_cr1^+^ cells were found (Fig. 5c and d). Given that *Cx_3_cr1* are highly expressed by YS-derived macrophages [34], Pax1-GFP^+^ Tnf^+^ macrophages are unlikely to have originated from YS. *In situ* immunofluorescence in sections across the E10.5 somite/aorta region confirmed the existence of Pax1-GFP^+^ Ter119^+^ erythrocytes (Fig. 5e) and Pax1-GFP^+^ CD68^+^/Arg1^+^/Tnf^+^ macrophages (Fig. 5f) within the aortic lumen. Immunofluorescence of single-cell smears, including intra-aortic blood cells, dissociated AGM cells and somite cells, from E10.5 embryos, followed by imaging quantification, also corroborated the predominance of Pax1-GFP^+^ Ter119^+^ erythrocytes and Tnf^+^/Arg1^+^/CD85K^+^ macrophages in the Pax1-GFP^+^ sIACs population (Fig. 5g).

### Contribution of mouse embryonic scHSPCs to long-term hematopoietic stem cells (LT-HSCs)

**Figure 6.**
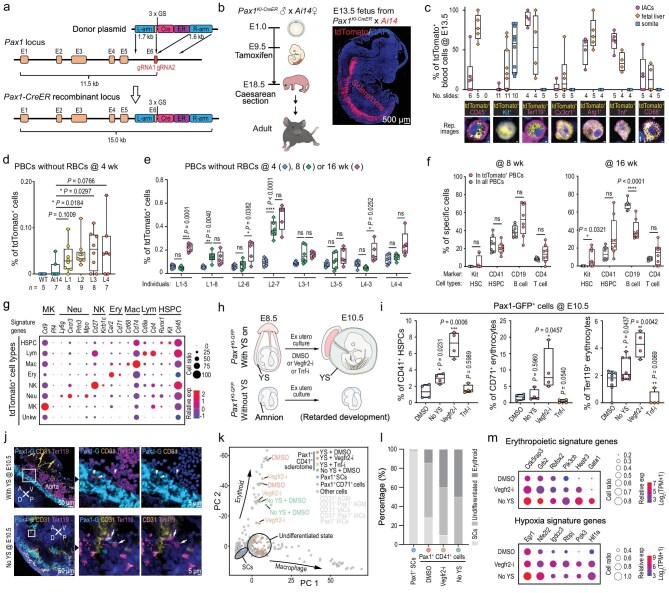
Characterization of sclerotome-derived hematopoietic cells in postnatal mice and their compensatory role for endothelial hematopoiesis. (a) Illustration of the *Pax1^KI-CreER^* knock-in locus. (b) Pax1^+^ lineage tracing strategy using Cre/tdTomato inducible system (left) and a typical fetus with sclerotomal tdTomato expression (right) after its mother was injected with 100 mg of tamoxifen at E9.5 and E10.5. (c) Ratio of cells with an indicated lineage marker in tdTomato^+^ fetuses; single-cell smears of two batches of multiple fetuses were immunostained and positive cells were counted on slides. (d) Ratio of tdTomato^+^ cells, revealed by flow cytometry, in PBCs of individual mice whose mothers were injected with tamoxifen at E9.5 during pregnancy (same as for e–g); *n*, number of tested animals; two-tailed *t*-test was performed (same as in e–g). (e) Ratio of tdTomato^+^ cells in PBCs of selected tdTomato^+^ mice as estimated by flow cytometry; for each litter (L), two individuals with higher ratio of tdTomato^+^ PBCs at 4 weeks old were selected for assays at later two ages (also for f and g). (f) Ratio of blood cells with lineage markers in tdTomato^+^ blood cells of selected tdTomato^+^ mice as estimated by flow cytometry. (g) Identification of specific hematopoietic blood cells from bone marrow of 10-month-old tdTomato^+^ mice; 192 tdTomato^+^ cells from eight mice were sequenced by Smart-seq2; lym, lymphocyte; mac, macrophage; ery, erythrocyte; neu, neutrophil; mk, megakaryocyte; unkw, unknown. (h) Experimental strategy for disrupting endothelial or YS hematopoiesis in *Pax1^KI-GFP^* embryos. (i) Ratio of Pax1-GFP^+^ IACs with lineage marker, revealed by flow cytometry; Vegfr2-i, Vegfr2 inhibitors; Tnf-i, Tnf inhibitor. (j) Representative images showing Pax1-GFP^+^ TER119^+^ cells in E10.5 embryos. Sections were immunoassayed. Pax1-GFP^+^ Ter119^+^ cells are indicated by arrowheads (top panel) or arrows (bottom panel). (k) PCA of cells from E10.5 embryos; the isolated cells were subjected to Smart-seq2 scRNA sequencing and the data were also used in (l) and (m). (l) Ratio of erythroid cells in Pax1^+^ CD41^+^ SCs population. (m) Bubble plot of expression levels of signature genes for indicated pathways in Pax1-GFP^+^ CD41^+^ cells.

For long-term tracking of the descendants of embryonic sclerotome-derived hematopoietic cells, we established a mouse *Pax1^KI-CreER^* line (Fig. 6a). When a *Pax1^KI-CreER^* male mates with an *Ai14* female harboring a loxP-Stop-LoxP-tdTomato cassette (JAX:007914), their progeny could continuously express the tdTomato reporter in SCs and their descendants after injection of tamoxifen into the mother to induce nuclear translocation of CreER and subsequent DNA recombination (Fig. 6b). Immunofluorescence of single-cell smears from tdTomato^+^ E13.5 fetuses whose mothers were injected with tamoxifen at E9.5 showed that large proportions of intra-aortic and fetal liver tdTomato^+^ cells were positive for the erythroid marker Ter119 and for the macrophage marker Tnf or Arg1, and that Kit^+^ tdTomato^+^ HSPCs existed in the fetal liver and somite region but were few in the aortic lumen (Fig. 6c). Sections and single-cell sequencing of E12.5 fetal liver also confirmed the existence of tdTomato^+^ cells and their hematopoietic identity (Fig. S6e and S6f). These observations are consistent with the finding in *Pax1^KI-GFP^* embryos that Pax1^+^ scHSPCs primarily contribute to the production of erythrocytes and macrophages during early development.

Next, we examined descendants derived from embryonic tdTomato^+^ SCs in mice after birth. Because of auto-fluorescence or the leaky effect of the LoxP system [36], we performed flow cytometry using PB of 4–8-week-old mice after removal of RBCs using several types of control mice (Fig. 6d, Fig. S7a). Flow cytometry analysis detected a significantly higher proportion of tdTomato^+^ blood cells in mice whose mothers were injected with tamoxifen during pregnancy compared to the control types of mice (Fig. 6d, Fig. S7a), though this was variable among litters. These observations imply that a certain proportion of PBCs with red fluorescence should have been descendants of Pax1-tdTomato^+^, sclerotome-derived HSPCs transformed during embryonic stages after tamoxifen induction. We chose individuals with a higher number of tdTomato^+^ blood cells from four litters (two individuals per litter) for flow cytometry using PBCs without RBCs at 4, 8 and 16 weeks old. Results showed a significant increase in the number of tdTomato^+^ blood cells in some individuals with age (Fig. 6e), implying that tdTomato^+^ blood cells most likely contain self-renewable HSCs. Flow cytometry following immunolabeling with specific lineage markers detected Kit^+^ HSCs, CD41^+^ HSPCs, CD4^+^ T cells and CD19^+^ B cells in PBCs or tdTomato^+^ PBCs at 8 and 16 weeks old from those eight tdTomato^+^ cell-containing mice (Fig. 6f). Furthermore, Smart-seq2 analysis of 192 tdTomato^+^ cells sorting from bone marrow of eight 10-month-old mice identified HSCs (14.06%), lymphocytes (12.5%), macrophages (22.92%), erythrocytes (19.27%), NK cells (10.94%), neutrophils (10.94%) and megakaryocytes (6.77%) (Fig. 6g). Taken together, these results support the idea that hematopoietic cells derived from Pax1^+^ SCs around E10 embryonic stages may contribute to the LT-HSC pool and be capable of differentiating into various blood lineages.

### Enhanced sclerotomal hematopoiesis in mouse embryos depleted in classical hematopoiesis

To investigate the hematopoietic function of sclerotome-derived blood cells, we disrupted endothelial hematopoiesis by intraperitoneally injecting a mixture of Vegfr2 signaling inhibitors (Brivanib + Foretinib + SU5408) [37–39] at E9.5 (Fig. S7b–e) or treating *ex utero* cultured *Pax1^KI-GFP^* embryos from E8.5 to E10.5 with a mixture of Vegfr2 inhibitors or depleted YS hematopoiesis by *ex utero* culturing embryos [40] after removal of YS (Fig. S7f–h, Fig. 6h–m). The control embryos at E10.5 appeared to have normal aorta anatomy and IAC emergence (Fig. S7b–e), robust blood circulation on YS with abundant erythrocytes and few blood cells within the embryonic body (Fig. S7f and S7g). In contrast, treatment with Vegfr2 inhibitors decreased the aorta perimeter (Fig. S7b and S7c), repressed IAC emergence (Fig. S7d), blocked YS circulation and resulted in possible generation of massive blood cells in the embryonic trunk (Fig. S7f, middle panel). Immunofluorescence revealed vessel disappearance and a lower number of CD41^+^ HSPCs on the YS of Vegfr2 inhibitor-treated embryos at E10.5 (Fig. S7g). However, although both aortic and YS endothelial hematopoiesis were impaired, sclerotome-derived blood IACs were not decreased (Fig. S7e). YS removal stunted embryonic development (Fig. S7f), but stimulated Ter119^+^ erythrocytes within the Pax1-GFP^+^ sclerotome at E10.5 (Fig. S7h). Flow cytometry using disassociated E10.5 embryonic cells revealed a higher number of Pax1-GFP^+^ HSPCs and erythrocytes in E10.5 *ex utero* embryos depleted in YS or treated with Vegfr2 inhibitors compared to control embryos (Fig. 6i). Immunofluorescence confirmed the sclerotomal origin of erythrocytes in the treated embryos (Fig. 6j). Tnf signaling has been shown to be required for aortic hematopoiesis [41]. We found that inhibition of the Tnf pathway using EVP4593 [42] reduced not only CD31^+^ but Pax1-GFP^+^ intra-aortic blood cells in either *in utero* or *ex utero* embryos (Fig. 6i), implying that all kinds of hematopoiesis may require Tnf signaling. Taking these data together, we hypothesize that, when YS or aortic hematopoiesis is interrupted, sclerotomal hematopoiesis is enhanced to meet the need for blood.

To further understand molecular changes of blood cells under the above stress conditions, we performed Smart-seq2 analysis of Pax1-GFP^+^ CD41^+^ cells (Table S5). Results showed that Pax1-GFP^+^ SCs could continue to contribute to hematopoietic blood cells expressing *Cd41* under Vegfr2 inhibition or YS removal conditions (Fig. 6k). Furthermore, the proportion of erythrocytes increased under these two conditions (Fig. 6l), being consistent with the flow cytometry results (Fig. 6i). In addition to remarkable up-regulation of erythropoietic signature genes (Fig. 6m, upper panel), the hypoxia signature genes *Hif1a, Pdk3, Rbpj, Igdcc3, Nfe2l2* and *Egr1* in both *ex utero* stress conditions were also up-regulated (Fig. 6m, lower panel) [43]. It is probable that YS removal or vessel interruption by Vegfr2 inhibition cause insufficiency of oxygen within the embryonic body, and as a result, the embryo has to enhance sclerotomal hematopoiesis to absorb oxygen. Like zebrafish sclerotomal hematopoiesis, therefore, mouse sclerotomal hematopoiesis may also help to maintain the robustness of hematopoiesis.

## DISCUSSION

In this study, we demonstrate that a subset of SCs in both zebrafish and mouse embryos are able to generate HSPCs in a temporal window during mid-somitogenesis stages (∼20–36 hpf in zebrafish, ∼E9–E10.5 in mice). This suggests that the embryonic sclerotome is an evolutionarily conserved hematopoietic tissue. Transdifferentiation of SCs into hematopoietic cells in zebrafish embryos seems totally independent of EHT, whereas this process in mouse embryos is mainly EHT-independent and also happens less frequently via EHT. Importantly, sclerotome-derived HSPCs may give rise to LT-HSCs in both species though they likely contribute much less to the HSC pool in adults compared to HSCs derived from endothelial hematopoiesis.

We noted that zebrafish scHSPCs in adults still express some somite/sclerotome-related genes except hematopoietic genes (Fig. 3d). The former may confer competency for differentiating into special non-hematopoietic cells during specific organ/tissue repair and regeneration processes. Transplanted embryonic scHSPCs in the zebrafish were able to repopulate and differentiate into versatile blood cell types. Unlike transplanted classical eHSPCs, interestingly, transplanted scHSPCs occasionally transform into muscle and fin rays (skeleton) in the zebrafish (Fig. S3c–l). This observation implies that fish scHSPCs may preserve non-hematopoietic multipotency.

Unlike in zebrafish, murine scHSPCs exhibit limited long-term potential and proliferate poorly in standard colony-forming unit (CFU) assays. This likely reflects their distinct biological properties, as conventional culture conditions optimized for classical HSPCs may not support the survival or expansion of scHSPCs. When tdTomato^+^ scHSPCs sorted from mouse intra-aortic blood or fetal liver were transplanted to X-ray-irradiated young mice, we were unable to recover the hematopoietic system of the hosts (data not shown). The possible causes include: (i) the number of scHSPC-derived LT-HSCs is too low in mice; (ii) mouse scHSPC-derived HSCs have poor self-renewal ability; and (iii) current transplantation assays may be inappropriate for evaluating certain embryo-derived stem cells, such as embryonic multipotent progenitors (eMPPs), which self-renew and differentiate physiologically but engraft poorly after transplantation [44]. We suggest that suboptimal donor cell preparation and unsuitable recipient niches may collectively compromise the survival of embryonic progenitors like scHSPCs in standard transplant settings.

Disruption of zebrafish endothelial hematopoiesis (via *runx1* mutation) triggers a compensatory increase in sclerotomal hematopoiesis, and simultaneous loss of both pathways (*runx1*/*nrp2b*) is lethal. These results suggest that two redundant hematopoietic pathways ensure robustness of hematopoiesis in fish. Although genetic studies in adult mice are limited by early embryonic lethality of *Runx1* knockout [45,46], pharmacological (Vegfr2 inhibition) or surgical (YS removal) impairment of circulation potently activates sclerotomal hematopoiesis in mouse embryos, confirming its conserved role as a backup. This compensatory response likely involves hypoxia sensing and altered niche signaling (e.g. cytokines) after endothelium deficiency. These mechanisms may together redirect sclerotomal progenitors toward hematopoiesis potential under stress. The exact underlying molecular mechanisms deserve further investigation.

## METHODS

Detailed Materials and Methods are available in the Supplementary data.

All animal procedures were performed in accordance with Tsinghua University's animal facility standards.

## Supplementary Material

nwag035_Supplemental_Files

## Data Availability

The raw sequence data reported in this paper have been deposited in the Genome Sequence Archive (Genomics, Proteomics & Bioinformatics 2025) in National Genomics Data Center (Nucleic Acids Res 2025), China National Center for Bioinformation/Beijing Institute of Genomics, Chinese Academy of Sciences (GSA: CRA035793) that are publicly accessible at https://ngdc.cncb.ac.cn/gsa.
