# Supplementary material for: Sclerotomal hematopoiesis in vertebrate embryos contributes to robustness of the blood system
Source: Natl Sci Rev. 2026 Jan 19;13(6):nwag035. doi: 10.1093/nsr/nwag035 (PMC13020419; doi:10.1093/nsr/nwag035)
Supplement: nwag035_Supplemental_Files [file nwag035_supplemental_files.zip › Supplementary_Data.docx]

**SUPPLEMENTARY INFORMATION FOR**

Sclerotomal hematopoiesis in vertebrate embryos contributes to robustness of the blood system

Zheng Jiang^1,2,3,†^, Tianqi Li^3,†^, Yixiao Song^2,3^, Luxi Chen^2,3^, Juhui Qiu^4^, Fan Zhou^3^,

Xiaotong Wu^2,3^, Jianbin Wang^3,*^, Anming Meng^1,2,3,*^

^*^Correspondence: [mengam@mail.tsinghua.edu.cn](mailto:mengam@mail.tsinghua.edu.cn) (A.M.) and [jianbinwang@tsinghua.edu.cn](mailto:jianbinwang@tsinghua.edu.cn) (J.W.)

**This document includes:**

Methods

Supplementary Figures S1-S7

Captions for supplementary Tables S1-S6 and Videos S1, S2.

**METHODS**

**Strains and transgenic lines**

WT zebrafish from the Tuebingen (Tu) strain were utilized. *Tg(-4.9kb-twist1a:Eos)* transgenic line was created by Dr. Qiu [12], and the *Tg(-3.5kb-ubiquitin-b:brainbow-1.0L)* line (*Zebrabow*) was generated using the Tol2-transposon system according to published research [47]. Transgenic lines including *Tg(fli1a:GFP)*, *Tg(kdrl:mCherry)*, *Tg(tal1:GFP)*, *Tg(coro1a:GFP)*, *Tg(mpx:GFP)*, *Tg(gata1a:GFP)* were maintained in our lab. *Tg(runx1:GFP)* was a gift from Feng Liu’s lab. *Tg(col1a2:Dendra2)* was a gift from Lingfei Luo’s lab. *nrp2a* and *nrp2b* mutants were engineered by co-injecting 1-cell stage WT embryos with 100 pg of gRNAs (synthesized using a MegaScript T7 kit (Ambion Cat # AM1334)) and 100 pg of Cas9 protein (NEB, Cat # M0386S) as detailed in Supplementary Table 6, to create founders. F1 progeny were PCR screened and sequenced for validation. The *runx1* mutant line (zko52b), with a 1bp insertion at the 164^th^ amino acid of exon4 causing a Runt domain frame shift, was developed by Dr. Feng Liu's lab [48]. Adult zebrafish for breeding were housed at 28.5℃ in a circulating system, with embryos raised in Holtfreter’s solution (0.059 M NaCl, 0.00067 M KCl, 0.00076 M CaCl_2_ and 0.0024 M NaHCO_3_), following Tsinghua University's animal care guidelines.

For mouse work, C57/B6J WT and *Ai14*/*LSL-tdTomato* (B6.Cg-Gt(ROSA)26Sortm14(CAG-tdTomato)Hze/J, JAX:007914) lines were obtained from Tsinghua University's animal facility or commercial vendors. Guide RNAs targeting *Pax1*'s 6th exon were synthesized using a MegaScript T7 kit. Donor plasmids for GFP and CreERT2 featured 1.7kb left and 1.6kb right homology arms, respectively, with constructs inserted downstream of the 6^th^ exon using a 3x GS linker. Pronuclear microinjection into C57B6/J zygotes involved 50 ng/μl Cas9 protein (NEB, Cat # M0386S), 10 ng/μl donor plasmid, and 50 ng/μl of each sgRNA. F_0_ founders were identified by PCR, and by microscopic observation of GFP expression for *Pax1^KI-GFP^* animals or of tdTomato expression for *Pax1^KI-CreER^* x *Ai14* embryos after tamoxifen (Sigma, Cat # 10540-29-1) induction (10 mg/mother intraperitoneally injected at E9.5). Mouse experimental protocols adhered to Tsinghua University's animal facility standards.

**Time-lapse imaging of zebrafish SC migration**

To observe SC migration, *Tg(twist1a:Eos)* was crossed with *Tg(fli1:GFP)* for dual labeling of early angioblasts and vasculature. Chorions of 20 hpf larvae from *Tg(twist1a:Eos;fli1:GFP)* were removed with pronase (Roche, Cat # 10165921001). The embryos were then placed laterally in grooves on a glass bottom dish (BeyoGold, Cat # FCFC016) with 0.75% low-melting point agarose (Amresco, Cat # 0815) for stability. A 3-D printed silver model with nine 0.7-mm semi-globes was pressed onto the agarose to create grooves. After cooling, the model was removed, leaving a thin agarose layer with grooves. Embryos were immobilized and positioned in the grooves under a microscope (Olympus FV1200) using tricaine (Ethyl 3-aminobenzoate methanesulfonate, Sigma, Cat # 886-86-2). The dish was transferred to a confocal microscopy chamber for imaging and UV irradiation. For photo-conversion of Eos from green to red in *Tg(twist1a:Eos)* embryos at 19-20 hpf, the ventral portion of 10-11 somites in the middle trunk region or all somites was irradiated with 405 nm UV light. After irradiation, embryos were imaged for about 10 hours at 28.5℃ using Olympus FV1200 confocal or Dragonfly spinning disk microscopy. Z-stack images were taken at 10-15 min intervals and 1-2µm Z intervals. The imaging field was adjusted every 2 hours. Traditional agarose embedment was avoided to prevent abnormal morphogenesis. For non-UV stimulated observation, *Tg(twist1a:Eos)* was crossed with *Tg(kdrl:mCherry)* following the same procedures.

To observe CHT homing of scHSPCs, embryos underwent similar procedures with photo-conversion at 20 hpf to label sclerotomes with red-Eos. After irradiation, embryos were returned to normal culture conditions shielded from light. At 36 or 48 hpf, labeled larvae were embedded laterally in 0.75% low-melting point agarose for imaging in the CHT region using the same microscopy setup. Holtfreter’s water with tricaine was added during live imaging to prevent larva movement while allowing heart beating and blood circulation.

**Ex vivo live imaging of mouse Pax1-GFP^+^ SC egression**

Ex vivo live imaging of E9.5 and E10.5 *Pax1^KI-GFP^* embryos was performed and adapted from Boisset et al.'s protocol [3, 49]. Initially, embryos had their head and tail removed, and the trunk was sectioned transversely into 100-200 μm slices, each containing about 2-3 pairs of somites, using a razor blade under a dissection microscope on a thermal plate at 37°C in PBS with 5% FBS. For lateral viewing of trunk truncation, the external surroundings of embryonic trunk after removal of head and tail were cleared, aiming to preserve the intra-aortic lumen as intact as possible, including the intra-aortic cells. Slices or truncated trunk were incubated with CD31-APC antibody (1:100, BioLegend, Cat # 102410) in DMEM and 20% homemade rat serum on ice for 30min, then placed in glass bottom wells and mounted in 0.3% low melting point agarose. Live imaging was conducted using a Nikon AX confocal system with a 25x Silicone oil lens and live cell conditions (37°C, 5% CO_2_). A reduced concentration of CD31-APC antibody (1:500) was used in the medium for continuous endothelial cell labeling over a 10-h imaging period. Z-stack images were performed with 1-2µm Z intervals and time series every 15-30 min. Unsectioned trunk regions were also imaged with focus on the ventral side of the aorta for a maximum imaging duration of 10 h to minimize artificial effects.

**Flow cytometry and cell sorting**

To collect blood from zebrafish transgenic larvae (>28 hpf), chorions were removed via pronase digestion, and larvae were anaesthetized with tricaine in a glass-bottom dish with blood buffer (10% FBS, 1 mg/ml heparin (Sigma, Cat # 9041-08-1) in PBS). Hearts were punctured under a dissection microscope to release blood into the buffer at room temperature, which was then filtered through a 40μm cell-strainer (Falcon, Cat # 352235) and chilled. For adult zebrafish blood, fish were anaesthetized with tricaine or ice-cold water, and blood was drawn from the tail cut and mixed with heparinized blood buffer. After peripheral blood collection, kidney marrow cells of the same fish were harvested by opening the belly, removing soft tissues, collecting the kidney, grinding, and filtering. Cell suspensions were stained with DAPI for dead cell exclusion before flow cytometry/sorting using BD FACSAria™ III.

To collect blood cells from mouse embryos, the head and tail of E10.5 *Pax1^KI-GFP^* and WT C57B6/J embryos were first removed, and then the trunk region was squeezed with tweezers from the middle to either end in PBS with 5% FBS at 37°C, allowing intra-aortic blood cells to flow into the medium. Blood cells were then collected and filtered through a 40μm cell-strainer. The aorta, gonad and mesonephros (AGM) were physically separated from surrounding tissues after removal of the head and tail, and the remaining trunk tissues were regarded as somite tissue, which actually contained spinal cord and intersegmental vessels with some blood cells. Embryonic tissues (AGM, somite etc.) were digested with 0.1% (W/V) collagenase IV (Sigma, Cat # C4-22-1G) at 37°C for < 30 min, and then filtered through a 40μm cell-strainer. Cells were incubated with Biolegend antibodies anti-mouse CD31 (Cat # 160209), CD41 (Cat # 133913), CD45 (Cat # 103112), Ter119 (Cat # 116212), Cx3cr1 (Cat # 149003), TNF-α (Cat # 506307), c-Kit (Cat # 161505), Ccr1 (Cat # 152503) with 1:100-1:200 dilutions in 10% rat serum PBS, and co-stained with DAPI for dead cell identification. The treated cells were analyzed/sorted using BD FACSAria™ III.

For adult mouse PB analysis, 3-5 μl blood was mixed with 500 μl 1x ACK lysis buffer (8.02 g NH_4_Cl, 1g KHCO_3_, 37.2 mg Na_2_EDTA in 850 mL of H_2_0) to lyse red blood cells for 10 min at room temperature. After centrifugation at 500g for 10 min, the cell pellet was resuspended in 10% rat serum PBS for antibody incubation on ice and flow cytometry.

**Fluorescent in situ hybridization**

Probe templates were PCR-amplified from specific-stage embryonic cDNAs using reverse primers with a T7 promoter (Supplementary Table S6). Digoxigenin- (Roche, Cat # 11277073910) or fluorescein- (Roche, Cat # 11685619910) labelled antisense RNA probes were synthesized in vitro using the mMESSAGE mMACHINE™ T7 Kit (Invitrogen AM1344). Whole-mount double fluorescent ISH (FISH) was performed and adapted from published method [50] using TSA plus fluorescence kits (Perkin Elmer, Cat # NEL744001KT and NEL745001KT). In brief, embryos were dechorionized by pronase, fixed in 4% PFA overnight, dehydrated in methanol, and rehydrated in PBST (PBS with 0.1% Tween-20) gradients. Embryos were pre-hybridized in HYB buffer (50% formamide, 5× SSC, 50 μg/mL yeast RNA, 50 μg/mL heparin, 0.1% Tween-20) at 65℃ for 1-2 h, then hybridized with antisense probes overnight at 65℃. After hybridization, embryos were washed with SSC, MABT, and blocked. Anti-fluorescein POD antibody (1:500) was applied overnight at 4℃ to label fluorescein probes. After washing with PBST, embryos were incubated in TSA cy3 solution (1:50) for 1-2 h at room temperature, followed by H_2_O_2_ treatment. A similar procedure was used for digoxin probes. Following extensive washing in 2x SSCT to reduce background fluorescence, embryos were DAPI-stained and mounted in 1.5% low-melting point agarose for confocal microscopy.

**Single-cell RNA-seq by Smart-seq2 and data analysis**

To collect single cell samples, zebrafish SCs of 19-hpf *Tg(twist1a:Eos)* were sorted through red-Eos after photo-conversion of green-Eos in sclerotomes. Blood cells at different stages were collected using the method described in the flow cytometry section. To collect blood cells from mouse embryos, the head and tail of E10.5 *Pax1^KI-GFP^* and WT C57B6/J embryos were first removed, and then the trunk region was squeezed with tweezers from the middle to either end in PBS with 5% FBS at 37°C, allowing intra-aortic blood cells to flow into the medium. Blood cells were then collected and filtered through a 40μm cell-strainer. The aorta, gonad and mesonephros (AGM) were physically separated from surrounding tissues after removal of the head and tail, and the remaining trunk tissues were regarded as somite tissue, which actually contained spinal cord and intersegmental vessels with some blood cells. Single cell suspensions were then immunostained with CD31-APC or CD45-APC antibodies (1:100) for cell sorting. E10.5 CD31^-^ Pax1-GFP^+^ SCs, CD45^+^ Pax1-GFP^+^ SCs, CD31^-^ Pax1-GFP^+^ IACs, CD31^+^ Pax1-GFP^+^ IACs, CD31^+^ Pax1-GFP^-^ IACs, CD31^+^ Pax1-GFP^+^ AGM/SCs, and CD31^+^ Pax1-GFP^-^ AGM cells were collected following single cell sorting. For *Pax1^KI-CreER^* × *Ai14* progenies, E12.5-E13.5 fetal liver, somite and intra-aortic cells were dissected and tdTomato^+^ cells were sorted. For ex-utero cultured embryos, Pax1-GFP^+^ CD41^+^ sSCs were collected by flow cytometry.

These single cells were individually placed into 200-µl PCR tubes with 2µl lysis including 0.2 % (vol/vol) Triton X- 100 and 2 U/µl RNase inhibitor (Thermo, Cat # N8080119) and stored at –80℃. To get full-length deep sequencing results [51], single-cell lysis, reverse transcription, cDNA pre-amplification and purification followed Smart-seq2 protocol [52]. The cDNA quality was checked on Agilent high-sensitivity DNA chip. cDNA libraries were made by TruePrep DNA Library Prep Kit V2 (Vazyme, Cat # TD501). Sequencing data was mapped to transcriptome by Salmon [53] with zebrafish Danio_rerio_GRCz11.92 reference or mouse Mm10 reference. PCA analysis was processed by Seurat V4 [54]. Trajectory analysis was performed by Monocle 2 [55]. Different expression genes (DEGs) analysis was processed by Deseq2 [56] and DEGs were used to perform GO Term analysis [57]. Heatmaps were generated by Microsoft Office Excel. Raw scRNA-seq data was deposited in the Genome Sequence Archive [58,59].

**High throughput single-cell RNA-seq by 10x genomics and data analysis**

Zebrafish blood cells from 28-hpf to 7-dpf larvae were collected as described in the flow cytometry section. For adult zebrafish, whole kidney marrow (WKM) was dissected and PB was collected from cutting tail after anaesthetization. RBCs in PB were lysed hypotonically [60] by mixing PB with an equal volume of blood buffer (PBS, 10% FBS, 1 mg/ml heparin), adding 10-fold volume of ice-cold ddH_2_O with 10-15 sec incubation, and then restoring osmolarity with 10× PBS. The mixture was filtered through a 40-μm cell-strainer. After DAPI staining, DAPI^+^ red blood cells were removed via FACS, and sorted cells were centrifuged at 600g for 5-10 min. Single cells were counted using a hemacytometer, and cDNA libraries were prepared with the Chromium Single Cell 3’ Library & Gel Bead Kit (10x genomics, Cat # PN-1000121), sequenced with novaseq6000. Sequencing data were aligned to the Danio_rerio_GRCz11.92 reference genome using Cellranger, and analyzed with Seurat V4 [53]. Standard quality filters for 10x datasets were: nCount_RNA > 1000 & < 20000, nFeature_RNA > 500 & < 3000, and mitoPercent < 10, with other parameters set to Seurat V4 defaults. Raw scRNA-seq data was deposited in the Genome Sequence Archive [58,59].

**scHSPCs transplantation and lineage tracing in zebrafish**

*Tg(twist1a:Eos)* were crossed with *Zebrabow* fish that express dTomato driven by the promoter of the housekeeping gene *ubiquitin*. At 20 hpf, embryos with high Eos and dTomato expression were selected. Eos; dTomato double-positive blood cells were sorted from 100-300 embryos at 28-30 hpf as described in the flow cytometry section. Sorted cells were centrifuged at 1000g for 5 min, resuspended in 2-3 μl blood buffer, and microinjected into the cardinal vein of 30-hpf WT larva, at about 20-30 nl (containing approximately 10-100 cells) per larva, using an air pressure system. Because some cells may be broken or have reduced viability due to damage during transplantation, the number of transplanted viable cells in a recipient was counted 12h post-transplantation by fluorescent microscopy. Then, recipients with more than 3 dTomato^+^ cells were raised for blood analysis and fluorescence tracing. GFP^+^; dTomato^+^ cells from embryos of *Tg(runx1:GFP)* x *Zebrabow* crosses served as control donors. For rescue assays, embryos from *runx1^+/-^*; *nrp2b^+/-^* fish intercrosses were genotyped at 1.5 dpf, and those with *runx1^+/-^*; *nrp2b^-/^*^-^, *runx1^+/-^*; *nrp2b^-/^*^-^, *or runx1^-/-^*; *nrp2b^-/^*^-^ genotype were used as recipients.

Transplanted larvae with dTomato^+^ cells in caudal hematopoietic tissue, thymus, fins, or kidney were monitored at various days post-fertilization (dpf). Adult recipients were also checked for dTomato fluorescence in muscle or fin, and some of them were sacrificed for PB and WKM analysis via flow cytometry or single-cell sequencing. To exclude cells with autofluorescence, only those cells with strong dTomato signal which were confirmed by PCR and single-cell sequencing transcripts were used for further analysis.

**Immunostaining and single cell imaging on mouse embryonic sections**

For embryonic sections, E10.5 embryos were immunostained with desired antibodies at 1:100 dilution in 10% FBS PBS on ice for 30min. The embryos were then fixed by 4% PFA for 1 h on ice. Immunostained embryos were embedded with opti-mum cutting temperature compound (OCT, SAKURA, Cat # 4583) for cryo-sections. Sections were then imaged by confocal microscopy.

For embryonic cell counting, E10.5 *Pax1^KI-GFP^* embryos or E13.5 *Pax1^KI-CreER^* x *Ai14* progenies whose mothers were pre-injected with 10 mg tamoxifen per individual at E9.5-E10.5 were dissected, and sclerotome, AGM, intra-aortic cells and fetal liver cells were collected. Cells were immunostained with Biolegend antibodies CD31 (Cat # 102520), Arginase I (Cat # 369705), TNF-α, Cx3cr1, CD68 (Cat # 137007), CD85k (Ilt3, Cat # 333015), Ter119, c-Kit, or CD45, all at 1:100 dilution and with Hoechest 33342 (Thermo, Cat # 62249) co-staining in 10% FBS PBS for 30 min on ice. Cells were then resuspended, placed on a glass bottom dish for confocal microscopic imaging with a 10x air lens at 2048px resolution. Hoechest signal facilitated whole cell counting using ImageJ's particle analysis (1-20 µm size threshold). Pax1-GFP and antibody double-positive cells were visually counted on each smear.

**Mouse Pax1-CreER lineage tracing and blood analysis**

*Pax1^KI-CreER^* males were mated to *Ai14* females. Females with virginal sperm plug were counted as E0.5. A pregnant female (about 20 grams) was intraperitoneally injected with 1 mg tamoxifen at E9.5 stage. Due to the toxicity of tamoxifen, the mothers usually had dystocia. Therefore, E18.5 pups were collected by caesarean section and raised up by ICR surrogacy. During caesarean section, pups were quickly dissected from the uterus and all slimes on the nose and mouth were cleaned. Healthy pups should then open the mouth with waving legs. Pups dissected from the same mother were kept in a 37°C incubator supplied with clean wet cotton before giving them to an ICR surrogate mother. The surrogate mothers were those that delivered their own babies about 1-2 days before. In our experiments, 8 pups were collected per litter on average. PB was taken for flow cytometry, starting from 4 weeks after caesarean section. tdTomato fluorescence and signals of desired antibodies (c-kit for HSCs, CD41 for HSPCs, Ter119 for erythrocytes, CD19 (Biolegend, Cat # 115511) for B cells, CD4 (Biolegend, Cat # 100411) for CD4 T cells) were checked as described above. Animals with higher number of tdTomato^+^ PBCs at 4 weeks old were bled again at 8 weeks and 16 weeks old for flow cytometry. Bone marrow from 8 tdTomato^+^ mice at 10 months old were also taken, and tdTomato^+^ cells were sorted for Smart-seq2 sequencing.

**Ex-uterus culture of embryos and related treatments**

Ex-utero culture was performed as previously reported [61]. In brief, E8.5 *Pax1^KI-GFP^* embryos were dissected from the uterus in dissection solution (FluoroBrite DMEM, Thermo, Cat # A1896701) with 10% homemade rat serum) using tweezer tip under a dissection microscope with a thermal plate at 37°C. The yolk sac should be intact. Then, embryos were ex-utero cultured in a rolling culture system (Precision Rodent Whole Embryo Culture System from Tanweizhiyan company, WEC001) supplied with 15% O_2_ for the first day in 2 ml culture medium (25% DMEM, 50% rat serum, 25% human cord blood serum and glucose). For control, embryos with yolk sac were incubated in culture medium with addition of 2.5 µl/ml DMSO. For Vegfr2 inhibition, embryos with yolk sac were incubated in culture medium in the presence of Vegfr2 inhibitors (Selleckchem SU5408, Cat # S6514, Fortinib, Cat # S1111, Brivanib, Cat # S1084, each 2.5µg/ml). For Tnf inhibition, embryos with yolk sac were incubated in culture medium in the presence of 10 µg/ml Tnf inhibitor EVP4593 (Selleckchem, Cat # S4902). For depleting the yolk sac, the yolk sac of embryos was carefully removed using a tweezer and then incubated in the culture medium containing 2.5 µl/ml DMSO. Rolling speed was set to 10 rpm. Pressure was set to extra 6-7 psi. Temperature was kept at 37°C. From E9.5 onwards, O_2_ proportion was set to 18%-21%. Additional glucose was added during daily culture medium change. E10.5 embryos were collected and analyzed by direct phenotype observation, flow cytometry or single cell sequencing as described in other sections.

**GO term and gene sets analysis**

GO term analysis was performed by Gene sets were downloaded from gsea-msigdb.org (UC SanDiego, https://www.gsea-msigdb.org/gsea/index.jsp). Gene sets used in this paper included: Riz Erythroid Differentiation (M1106) [62] and Reactome Signaling by Erythropoietin (MM15584). GSEA analyses were performed using GSEA applications from GSEA official site (GSEA v4.3.2 Mac App). Signature genes of hypoxia pathway and erythropoiesis were selected from gene sets for bubble plotting. The bubble area showed proportion of cells expressing specific genes (TPM > 0) in that group. The bubble color scale showed log2 average expression level (TPM + 1) of specific genes in that group.

**Image processing**

The time-lapse multiple-view z-stack images of embryos were processed using Imaris software version 9.3 or higher (Bitplane AG). Contrast and brightness were adjusted in Imaris to clearly show cell morphology. Regions of interest were cropped using Imaris crop 3D option to do 3D reconstitution using surface function. All imaging photos were assembled in Adobe Photoshop 2020.

**Quantification and statistical analysis**

Statistical analyses and plots were performed with Microsoft Excel, Prism 9. Most statistical analyses used mean ± SEM unless specifically stated in figure legends. Significance between different samples were carried out using unpaired, two-tailed *t*-tests. *P* values were indicated specifically in figures or legends.

**REFERENCES of METHODS**

1. Pan YA, Freundlich T and Weissman TA et al. Zebrabow: multispectral cell labeling for cell tracing and lineage analysis in zebrafish. Development 2013; **140**: 2835–46.
2. Sun Y, Zhang B and Luo L et al. Systematic genome editing of the genes on zebrafish Chromosome 1 by CRISPR/Cas9. Genome Res 2020; **30**: 118–26.
3. Boisset JC, Andrieu-Soler C and van Cappellen WA et al. Ex vivo time-lapse confocal imaging of the mouse embryo aorta. Nat Protoc 2011; **6**: 1792–805.
4. Brend T and Holley SA. Zebrafish whole mount high-resolution double fluorescent in situ hybridization. J Vis Exp 2009; **25**: 1229.
5. Sun F, Li H and Sun D *et al.* Single-cell omics: experimental workflow, data analyses and applications. *Sci China Life Sci* 2025; **68**: 5–102.
6. Picelli S, Faridani OR and Bjorklund AK et al. Full-length RNA-seq from single cells using Smart-seq2. Nat Protoc 2014; **9**: 171–81.
7. Patro R, Duggal G and Love MI et al. Salmon provides fast and bias-aware quantification of transcript expression. Nat Methods 2017; **14**: 417–9.
8. Hao Y, Hao S and Andersen-Nissen E et al. Integrated analysis of multimodal single-cell data. Cell 2021; **184**: 3573–87.
9. Qiu X, Mao Q and Tang Y et al. Reversed graph embedding resolves complex single-cell trajectories. Nat Methods 2017; **14**: 979–82.
10. Love M, Huber W and Anders S. Moderated estimation of fold change and dispersion for RNA-seq data with DESeq2. Genome Biol 2014; **15**: 550.
11. Yu G, Li F and Qin Y et al. GOSemSim: an R package for measuring semantic similarity among GO terms and gene products. Bioinformatics 2010; **26**: 976–8.
12. Zhang S, Chen X and Jin E *et al.* The GSA Family in 2025: A Broadened Sharing Platform for Multi-omics and Multimodal Data. *Genomics Proteomics Bioinformatics* 2025; **23**: 100772.
13. CNCB-NGDC Members and Partners. Database Resources of the National Genomics Data Center, China National Center for Bioinformation in 2025. *Nucleic Acids Res* 2025; **53**: D30–D44.
14. Hu Y, Maisey K and Subramani PA et al. Characterisation of rainbow trout peripheral blood leucocytes prepared by hypotonic lysis of erythrocytes, and analysis of their phagocytic activity, proliferation and response to PAMPs and proinflammatory cytokines. Dev Comp Immunol 2018; **88**: 104–13.
15. Aguilera-Castrejon A, Oldak B and Shani T et al. Ex utero mouse embryogenesis from pre-gastrulation to late organogenesis. Nature 2021; **593**: 119–24.
16. Riz I, Akimov SS and Eaker SS et al. TLX1/HOX11-induced hematopoietic differentiation blockade. Oncogene 2007; **26**: 4115–23.


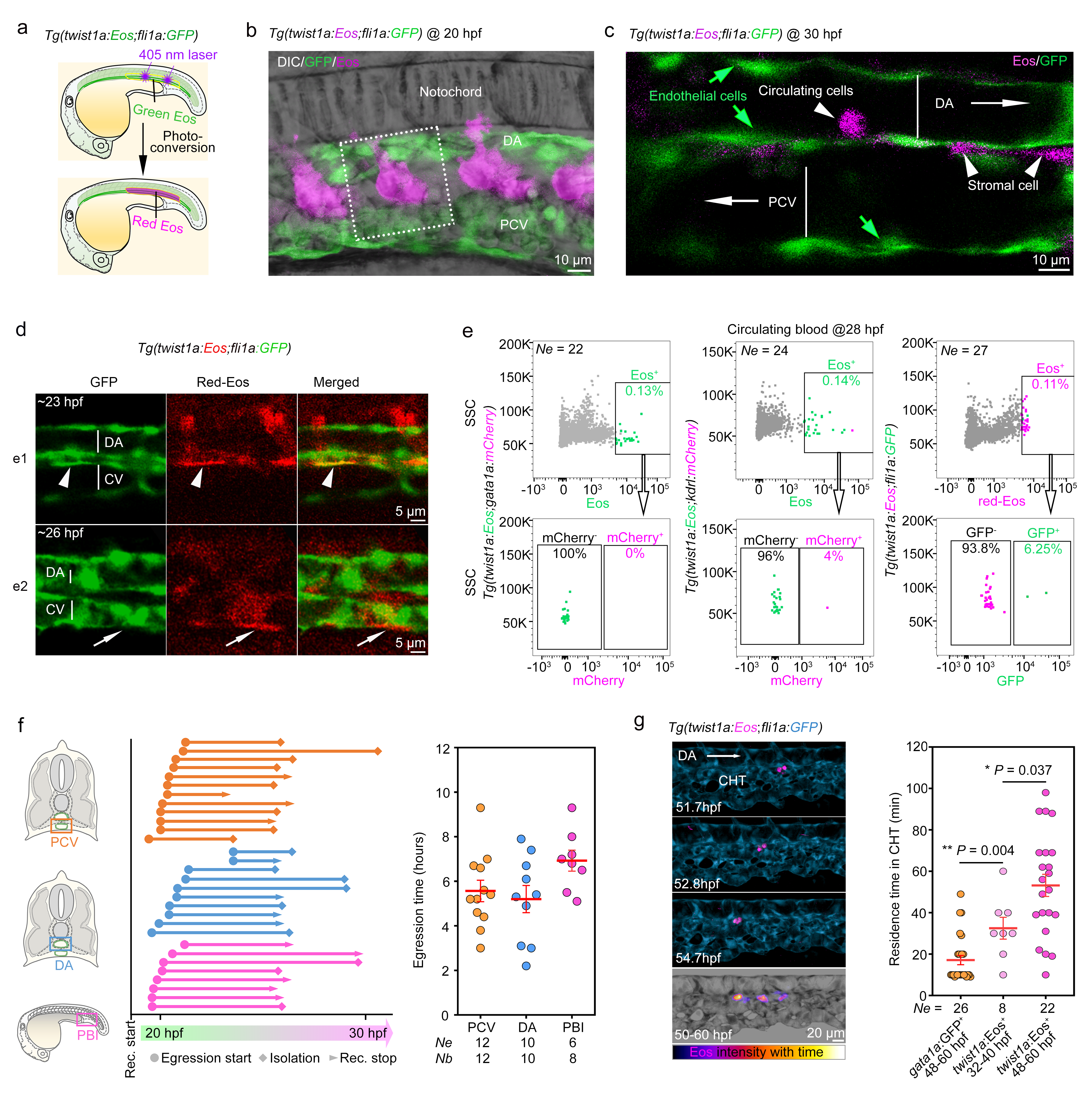


**Figure S1. Expression of the reporter Eos in zebrafish *Tg(twist1a:Eos)* transgenic embryos, related to Figure 1.**

(a) Schematic diagram of the strategy for UV-irradiating sclerotome compartments of 10-11 somites of a *Tg(twist1a:Eos)* transgenic embryo at 19-20 hpf. (b) Confocal microscopic images of a 4-somite segment in a *Tg(twist1a:Eos;fli1a:GFP)* embryo at 20 hpf after UV-irradiation. Eos was converted from green to red (shown in magenta). Endothelial cells were labeled by *fli1a*:GFP; white dashed box contained a single cluster of sclerotomal cells; DA, dorsal aorta; PCV, posterior cardinal vein. (c) Lateral view of trunk DA and PCV in a *Tg(twist1a:Eos;fli1a:GFP)* at 30 hpf. All somites were UV-irradiated at 19 hpf. Sclerotome-derived cells in red; endothelial cells were labeled by *fli1a*:GFP (green). (d) Confocal microscopic images of two *Tg(twist1a:Eos;fli1a:GFP)* embryos. Embryos were UV-irradiated at 19 hpf and observed at indicated stages. In e1, one endothelial cell (indicated by an arrowhead) co-expressed *fli1a*:GFP and *twist1a*:Eos; in e2, the arrow-indicated cell may also be an endothelial cell. (e) Flow cytometry analysis of 28-hpf *twist1a*:Eos*^+^* blood cells from indicated double transgenic embryos. Lower panels showed the ratio of *gata1a^+^*, *kdrl^+^* or *fli1a^+^* cells among Eos^+^ blood cells. (f) Schematic diagram showing the timeline of sclerotomal cell emigration process based on live imaging records. Statistic results showed duration of time for emigration of sclerotomal cell into the blood vessel (right). Ne, number of observed embryos; Nb, number of observed blood cells. (g) Residence time of sclerotome-derived blood cells in CHT at earlier stages compared with *gata1a*:GFP^+^ blood cells that do not normally resident in CHT).


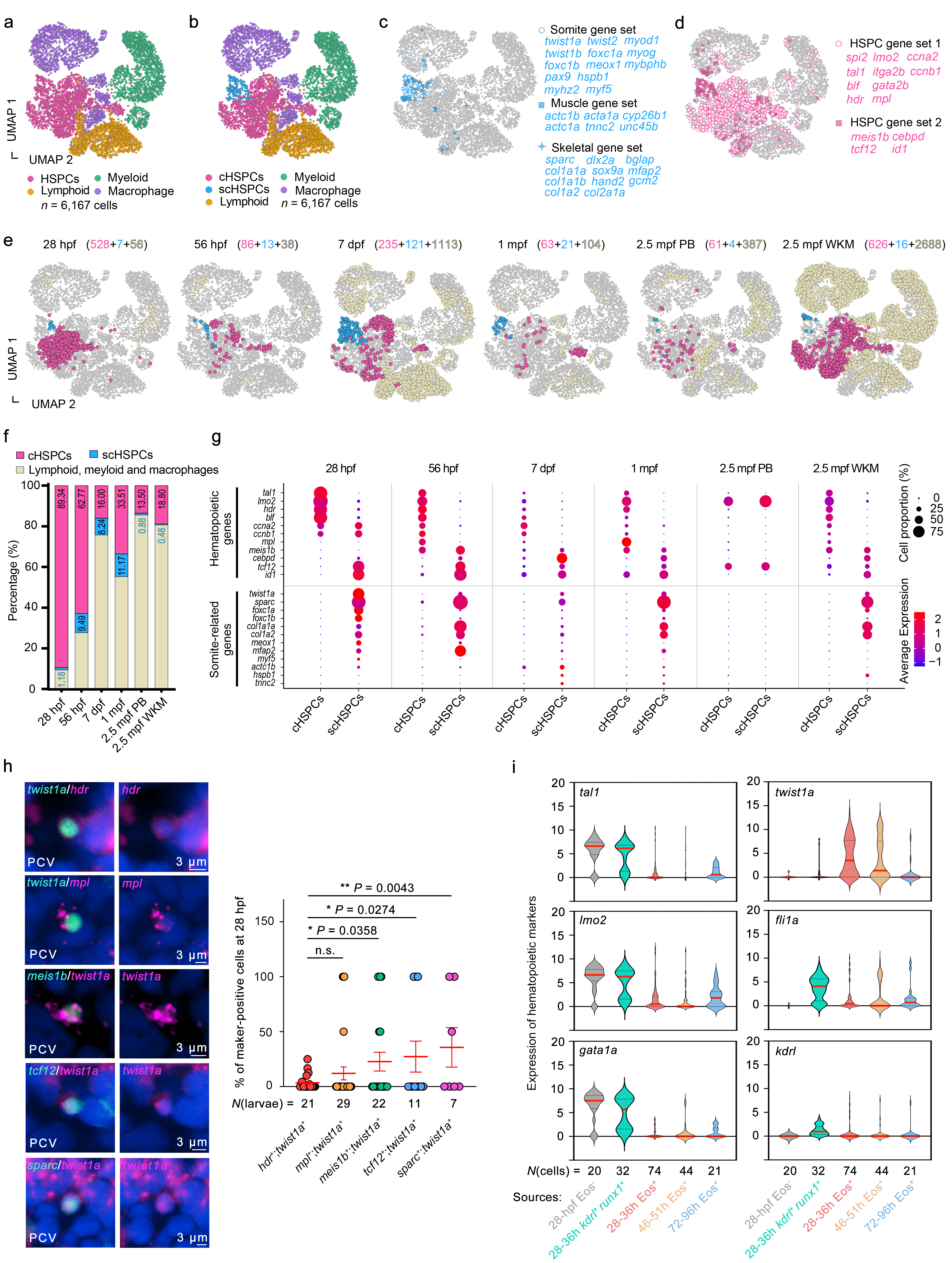


**Figure S2. Identification of somite-derived HSPCs at single-cell level in the zebrafish, related to Figure 2.**

(a) UMAP clustering of integrated blood cells of WT embryos/fish at 28 hpf, 7 dpf, 1 mpf and 2.5 mpf; different types of cells were represented by different colors; *n*, total number of analyzed cells. (b) UMAP clustering of blood cells, being similar to (a) except that the HSPCs population was divided into two subpopulations: scHSPCs and cHSPCs. (c and d) Highlights of cells with high expression levels of indicated gene sets on the integrated UMAP (b); genes with normalized expression level > 0.6 (maximum set to 1.0) were selected only; HSPC gene sets 1 and 2 in (d) were expressed in cHSPCs and scHSPCs, respectively. (e) Highlights of cHSPCs (red) and scHSPCs (blue) and other cells (beige) on the integrated UMAP at different stages; cell number for each cell type was indicated in parenthesis. (f) Ratios of different blood cell types at different stages. (g) Bubble plot of representative hematopoietic genes and somite-related genes expression in cHSPCs and scHSPCs. (h) Co-detection of sclerotome gene *twist1a* with the hematopoietic markers by FISH in PCV at 28 hpf. Markers: *hdr and* *mpl*, classical hematopoietic markers; *meis1b* and *tcf12,* scHSPC-expressing hematopoietic markers; *sparc,* skeletal marker. Left, representative FISH images; right, the proportion of cells co-expressing *twist1a* and an indicated maker to the total number of *twist1a*-expressing cells. *N*(larvae), number of observed larvae. (i) Expression of the sclerotome gene *twist1a*, the well-known lateral plate mesoderm genes *tal1*, *lmo2* and *gata1a*, and the endothelial genes *fli1a* and *kdrl* in blood cells at various stages. Data were extracted from single cell Smart-Seq2 and expressed as log_2_(TPM+1). 28-hpf *twist1a*:Eos^-^ blood cells represent primitive blood cells, *twist1a*:Eos^+^ (Eos^+^) cells represent sclerotome-derived blood cells, *kdrl*:mCheery; *runx1*:GFP double-positive (*kdrl^+^runx1^+^*) blood cells represent definitive blood cells generated via endothelial to hematopoietic transition. *N*(cells), number of observed cells.


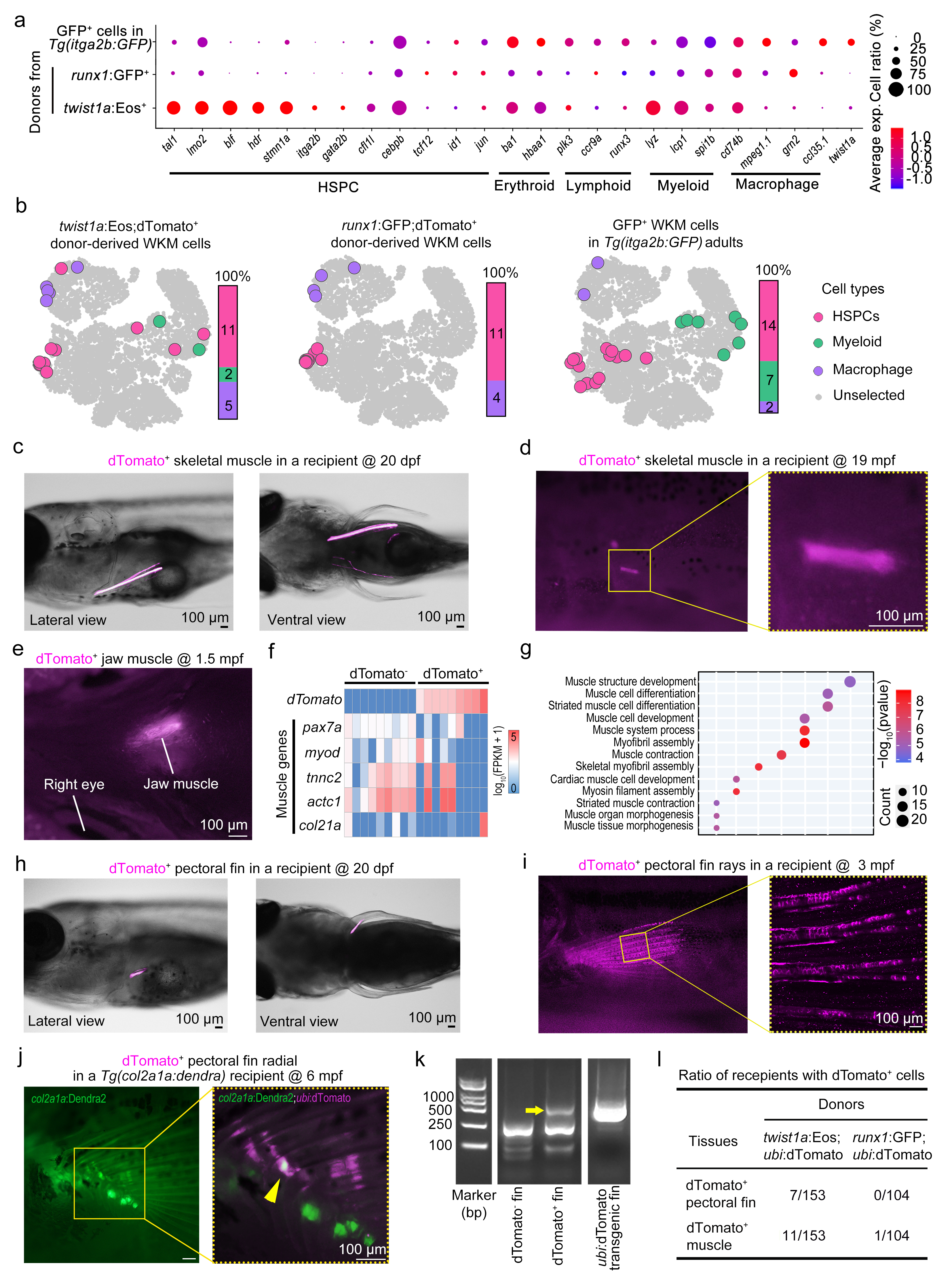


**Figure S3. Hematopoietic and nonhematopoietic differentiation of zebrafish transplanted scHSPCs, related to Figure 2.**

(a) Hematopoietic marker gene expression in different types of donor hematopoietic derivatives in WKM of adult recipients based on Smart-seq2 data. (b) Projection of donor-derived WKM blood cells to WT blood UMAP; sorted dTomato^+^ single cells were sequenced and cells without dTomato transcripts were filtered. (c-e) Distinguishable dTomato^+^ muscle derived from transplanted dTomato^+^ scHSPCs in recipients; the stronger fluorescent muscle fibers looked opaline because of too strong fluorescence. (f) Heatmap of typical muscle genes expression in single cells; a cell mass surrounding dTomato^+^ muscle-like cells in several recipients was dug up and disassociated for Smart-seq2 scRNA seq; cells were divided into dTomato^-^ and dTomato^+^ cells according to scRNA seq profiles. (g) GO term function prediction of dTomato^+^ muscle cells based on single cell RNA seq data. (h and i) Representative dTomato^+^ fin in recipients at indicated ages. (j) a dTomato^+^ distal radial in a *Tg(col2a1a:Dendra2)* recipient at 6 mpf; Denra2 marked distal radials of the pectoral fin. (k) Confirmation of the dTomato gene by PCR in dTomato^+^ pectoral fin of recipients at 3 months old; the positive band of 515 bp was indicated by an arrow. (l) Summary of transplantation results.


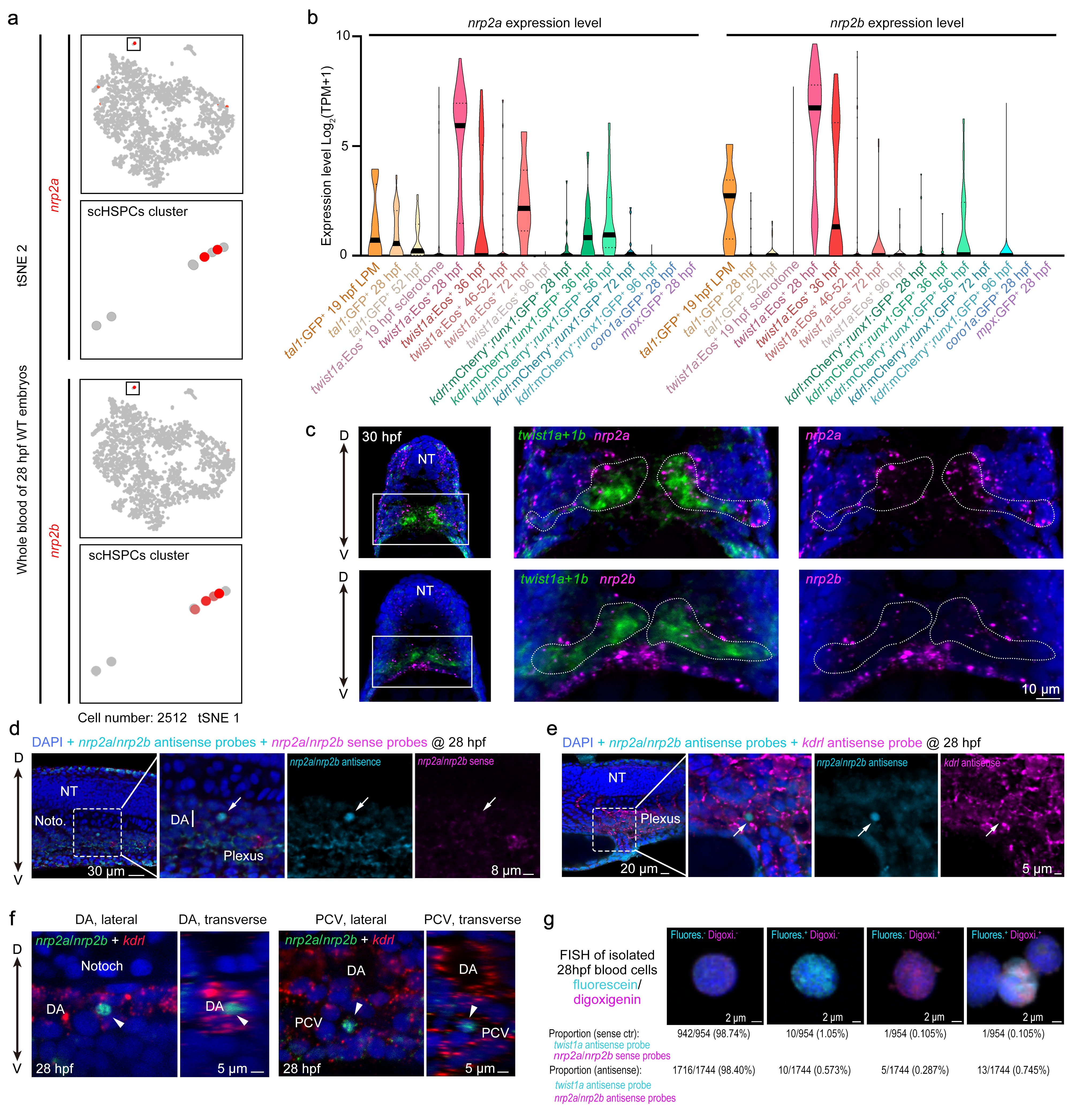


**Figure S4. scHSPC-specific expression of zebrafish *nrp2a* and *nrp2b*, related to Figure 3.**

(a) tSNE clustering of blood cells derived from embryos at 28 hpf following 10x genomics single-cell RNA sequencing; the scHSPCs cluster was marked with a rectangular box (left panel) and zoomed in (right panel), and cells with *nrp2a* and *nrp2b* expression were displayed in red color. (b) Expression level (Log_2_(TPM+1)) of *nrp2a* and *nrp2b* in cells of different origins; cells isolated from indicated transgenic embryos at 19-96 hpf were subjected to Smart-seq2 single-cell RNA sequencing. The transgenic reporter-labeled cell types: *tal1*, primitive HSPCs; *kdrl*, cHSPCs; *runx1*, cHSPCs; *twist1a*, scHSPCs; *coro1a*, macrophage; *mpx1*, neutrophil. (c) *nrp2a* and *nrp2b* were co-expressed with *twist1a*/*twist1b* in some SCs; embryos at 20 hpf were subjected to FISH and observed by confocal microscopy after cross cryosectioning; the *twist1a*/*twist1b*-labeled sclerotome was marked. (d) Representative FISH images showing *nrp2a*/*nrp2b* expression in a single blood cell (indicated by an arrow) in dorsal aorta (DA) of a 28-hpf WT embryo. FISH with *nrp2a*/*nrp2b* sense probes served as control. (e) Representative FISH images showing *nrp2a*/*nrp2b* expression in a single blood cell (indicated by an arrow) in posterior vein plexus (labeled by *kdrl* antisense probe) of a 28-hpf WT embryo. (f) Representative FISH images showing *nrp2a*/*nrp2b* expression in single blood cell (indicated by arrowheads) in DA and PCV of 28-hpf WT embryos. The endothelial cells were detected with *kdrl* antisense probe. (g) Counting of blood cells co-expressing endogenous *twist1a* and *nrp2a*/*nrp2b* in isolated 28-hpf blood from WT larvae. *nrp2a*/*nrp2b* sense probes as negative control.


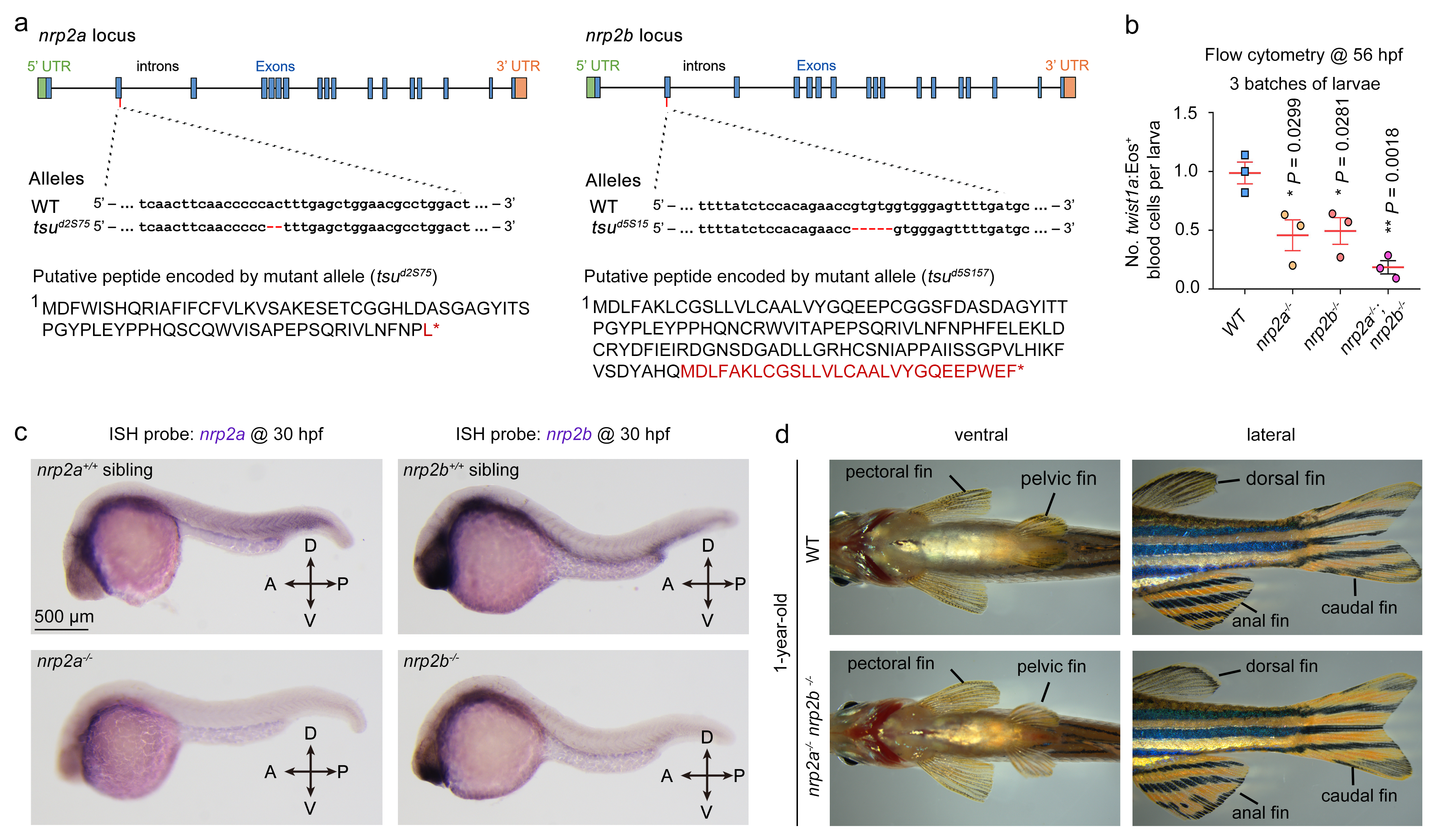


**Figure S5. Genetic mutations of zebrafish *nrp2a* and *nrp2b*, related to Figure 3.**

(a) Generation of *nrp2a* and *nrp2b* mutant lines by CRISPR/CAS9; top, the genomic structure of the indicated gene; middle, DNA sequence flanking deleted nucleotides; bottom, predicted mutant allele-encoded peptide sequence with changed amino acids in red. (b) Number of *twist1a*:Eos^+^ scHSPCs in 56-hpf larvae with different genotypes; WT, siblings (*nrp2b^+/+^*) of *nrp2b^-/-^* mutants; all types of larvae harbored and expressed the *Tg(twist1a:Eos)* transgene; blood cells taken from hearts of 20-30 larvae with the same genotype were subjected to flow cytometry for counting *twis1a*:Eos^+^ cells; the average number per larva was shown; three batches of larvae for each genotype served as biological replicates; two-tailed *t*-test was performed. (c) *nrp2a* and *nrp2b* transcripts were detected by WISH in somites and other tissues of wild-type siblings and mutant embryos at 30 hpf. Embryos were laterally viewed with indicated anteroposterior and dorsoventral axes. (d) Fin phenotype in one-year-old wild-type and *nrp2a*/*nrp2b* double mutants.


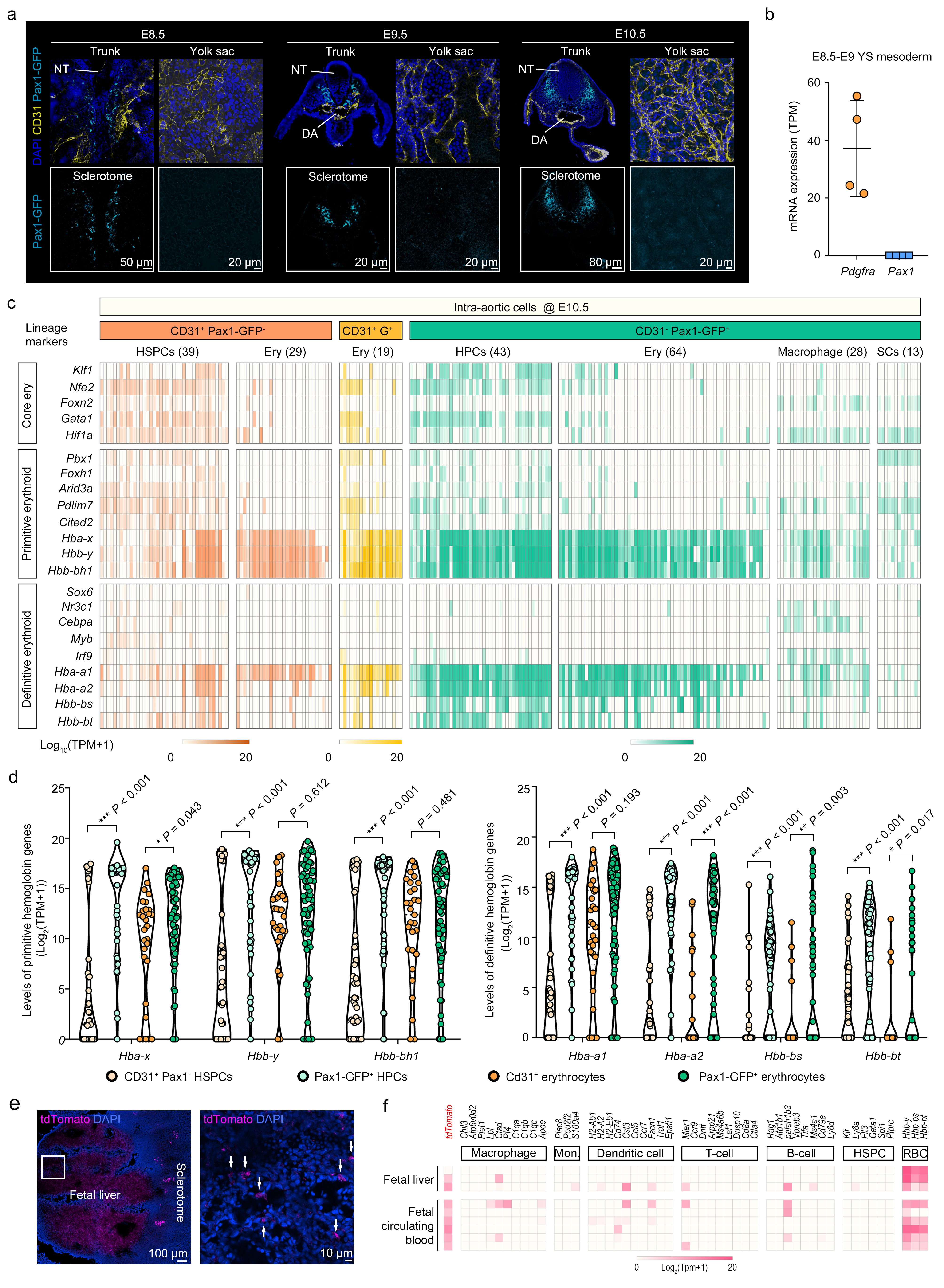


**Figure S6. Contribution of Pax1^+^ sclerotomal cells to hematopoietic lineages in mouse embryos, related to Figure 5.**

(**a**) Cross sections showing sclerotome-specific GFP expression in E8.5-E10.5 *Pax1^KI-GFP^* intra-embryonic trunk but not in extra-embryonic yolk sac. Blood vessels were indicated by the endothelial marker CD31 expression. NT, neural tube; DA, dorsal aorta. (**b**) Gene expression levels of the mesoderm marker *Pdgfra* and *Pax1* in E8.5-E9 yolk sac cells by bulk-RNA sequencing. (**c**) Heatmap of expression levels of erythroid genes in different types of intra-aortic cells; the number of cells was shown in parenthesis; CD31^+^ G^+^, CD31^+^ Pax1-GFP^+^; HSPCs, hematopoietic stem and progenitor cells; HPCs, hematopoietic progenitor cells; Ery, erythrocytes; SCs, sclerotomal cells. (**d**) Expression levels of primitive and definitive hemoglobin genes in different types of intra-aortic cells; two-tailed *t*-test was performed. (**e**) Representative image of a liver section of E12.5 *Pax1^KI-CreER^* fetus after induction with 1 mg tamoxifen at E9.5. The boxed area in the left image was enlarged on the right. tdTomato^+^ cells were indicated by arrows. (**f**) Confirmation of co-expression of *tdTomato* with blood markers in tdTomato^+^ cells sorting from E12.5 liver or circulating blood of *Pax1^KI-CreER^* fetuses (E9.5 tamoxifen induction) by single cell Smart-Seq2.


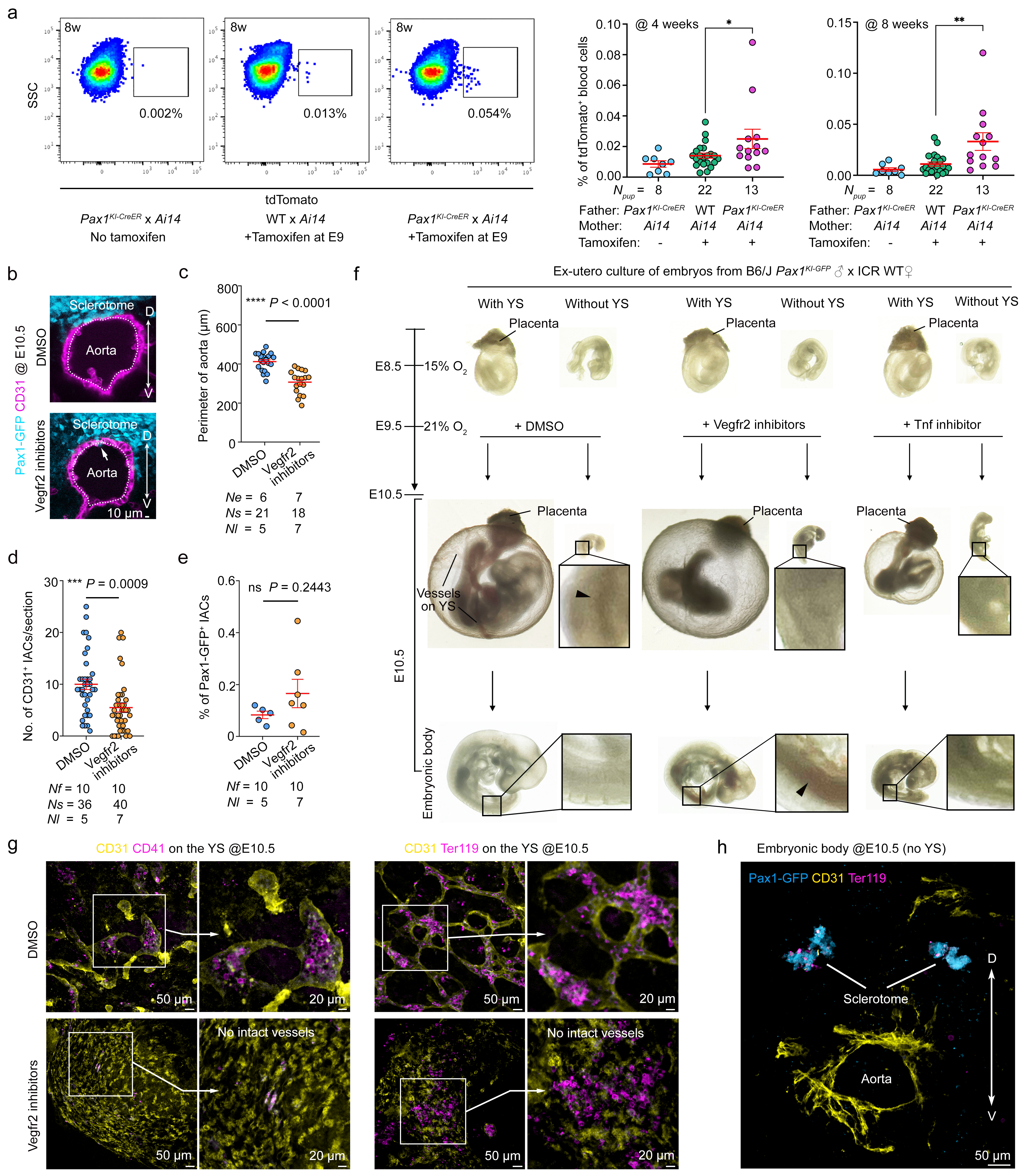


**Figure S7. Specificity confirmation of tamoxifen-induced tdTomato^+^ blood cells and effect of Vegfr2 signaling inhibition or yolk sac removal on embryonic hematopoiesis, related to Figure 6.**

(**a**) Analysis of tdTomato^+^ peripheral blood cells by flow cytometry. Peripheral blood was taken from 4-wk and 8-wk pups whose mothers were treated or untreated with tamoxifen at E9.0. Pups from Ai14×WT crosses with E9 tamoxifen induction were also used as controls. *N_pup_*, number of observed pups. (**b-e**) Effect of Vegfr2 signaling inhibition on aorta, IACs and Pax1-GFP^+^ sIACs generation. DMSO (100 μg/mother), Vegfr2 inhibitors (SU5408 + Foretinib + Brivanib, 100 μg each/mother) was delivered into *Pax1^KI-GFP^* mothers by intraperitoneal injection at E9.5. At E10.5, embryos were sectioned and stained with CD31-APC antibody. Representative images were shown in (**b**) with Pax1-GFP^+^ sIAC indicated by arrow. The perimeter of the aortic lumen (**c**) and the number of CD31^+^ IACs (**d**) were counted on sections after imaging. The ratio of Pax1-GFP^+^ sIACs (**e**) were counted by flow cytometry. *Ne*, number of observed fetuses; *Ns*, number of observed sections, *Nl*, number of litters. (**f**) Morphological changes in ex utero cultured *Pax1^KI-GFP^* embryos under different conditions; blood clump in the embryo body was indicated by arrowhead in embryos without yok sac (YS) or embryos with YS but in the presence of Vegfr2 inhibitors (SU5408 + Foretinib + Brivanib, each 2.5 μg/ml); the Tnf inhibitor EVP4593 was applied at a concentration of 10 μg/ml. (**g**) Effect of Vegfr2 inhibitors treatment on CD41^+^ HSPCs and Ter119^+^ erythrocytes on the YS; the embryos with YS were co-immunostained with the endothelial marker CD31 and the hematopoietic marker CD45 or Ter119, and observed by confocal microscopy with focus on the YS. (**h**) Appearance of Ter119^+^ erythrocytes in the sclerotome region in an embryo ex utero cultured without YS.

**Table S1.**

Smart-seq2 gene expression matrix of labeled cells in zebrafish transgenic embryos, **related to Figure2.**

**Table S2.**

Smart-seq2 gene expression matrix of zebrafish blood cells in kidney marrow, **related to Figure2 and Figure S3.**

**Table S3.**

Smart-seq2 gene expression matrix of selected muscle cells in zebrafish, **related to Figure S3.**

**Table S4.**

Smart-seq2 gene expression matrix of mouse E10.5 embryonic cells, **related to Figure 5.**

**Table S5.**

Smart-seq2 gene expression matrix of ex-utero cultured mouse embryonic cells, **related to Figure 6.**

**Table S6.**

Primer sequences used in this study**.**

**Video S1, related to Figure 1.**

Showcase of a zebrafish sclerotomal cell (*twist1a*:Eos^+^, photoconverted at 20 hpf, magenta color, white arrowhead indicated) egression into PCV (labeled by *fli1a*:GFP) lumen from 21 hpf. Lateral view (left panel) and transverse view were demonstrated.

**Video S2, related to Figure 1.**

Video showing zebrafish scHSPCs (*twist1a*:Eos^+^, photoconverted at 20 hpf, magenta color, white arrow and arrowhead indicated) residence in CHT (endothelium labeled by *fli1a*:GFP). The second scHSPC (white arrow) was undergoing mitosis and produced two daughter cells (white arrows) during residence in CHT. The daughter cells left CHT after mitosis (dashed circle line).
